# Design, Synthesis, and Biological Evaluation of Novel 3-Cyanopyridone/Pyrazoline Hybrids as Potential Apoptotic Antiproliferative Agents Targeting EGFR/BRAF^V600E^ Inhibitory Pathways

**DOI:** 10.3390/molecules28186586

**Published:** 2023-09-12

**Authors:** Lamya H. Al-Wahaibi, Hesham A. Abou-Zied, Mohamed Hisham, Eman A. M. Beshr, Bahaa G. M. Youssif, Stefan Bräse, Alaa M. Hayallah, Mohamed Abdel-Aziz

**Affiliations:** 1 Department of Chemistry, College of Sciences, Princess Nourah bint Abdulrahman University, Riyadh 11564, Saudi Arabia; lhalwahaibi@pnu.edu.sa; 2 Medicinal Chemistry Department, Faculty of Pharmacy, Deraya University, Minia 61111, Egypt; drhesham92@yahoo.com (H.A.A.-Z.); mohammedhisham90@yahoo.com (M.H.); 3 Medicinal Chemistry Department, Faculty of Pharmacy, Minia University, Minia 61519, Egypt; emanbeshr@yahoo.com (E.A.M.B.); abulnil@hotmail.com (M.A.-A.); 4 Pharmaceutical Organic Chemistry Department, Faculty of Pharmacy, Assiut University, Assiut 71526, Egypt; 5 Institute of Biological and Chemical Systems, IBCS-FMS, Karlsruhe Institute of Technology, 76131 Karlsruhe, Germany; 6 Pharmaceutical Organic Chemistry Department, Faculty of Pharmacy, Sphinx University, Assiut 71515, Egypt

**Keywords:** pyridine, pyrazoline, synthesis, anticancer, apoptosis, docking

## Abstract

A series of novel 3-cyanopyridone/pyrazoline hybrids (**21–30**) exhibiting dual inhibition against EGFR and BRAF^V600E^ has been developed. The synthesized target compounds were tested in vitro against four cancer cell lines. Compounds **28** and **30** demonstrated remarkable antiproliferative activity, boasting GI_50_ values of 27 nM and 25 nM, respectively. These hybrids exhibited dual inhibitory effects on both EGFR and BRAF^V600E^ pathways. Compounds **28** and **30**, akin to Erlotinib, displayed promising anticancer potential. Compound **30** emerged as the most potent inhibitor against cancer cell proliferation and BRAF^V600E^. Notably, both compounds **28** and **30** induced apoptosis by elevating levels of caspase-3 and -8 and Bax, while downregulating the antiapoptotic Bcl2 protein. Molecular docking studies confirmed the potential of compounds **28** and **30** to act as dual EGFR/BRAF^V600E^ inhibitors. Furthermore, in silico ADMET prediction indicated that most synthesized 3-cyanopyridone/pyrazoline hybrids exhibit low toxicity and minimal adverse effects.

## 1. Introduction

Cancer is a leading cause of death [1,2]. Cancer has the ability to invade and harm healthy tissues and disrupt functions; metastasis worsens treatment prospects [3,4]. Early detection, prevention, and enhanced treatment are vital to combat cancer’s impact on individuals and society [5,6]. Chemotherapy damages rapidly dividing cells like bone marrow, digestive tract, and hair follicle cells, causing side effects like hair loss, nausea, fatigue, and infection vulnerability [7]. Targeted treatments offer personalized, less harmful cancer care than traditional chemotherapy [8,9,10]. Tailored to cancer type and molecular traits, they enable precise and personalized therapy.

The EGFR (Epidermal Growth Factor Receptor) is crucial in cancer due to its frequent overexpression or mutations [11,12]. EGFR targeting is prominent in cancer treatment, with TKIs and monoclonal antibodies designed to disrupt signaling and reduce proliferation [13,14]. These targeted therapies have shown clinical success in several cancer types, including lung, colorectal, and head and neck cancer [15,16,17]. EGFR-targeted therapy faces resistance through diverse mechanisms, underscoring the need for ongoing research to enhance its effectiveness and counter resistance [18]. BRAF is pivotal in cancer treatment, especially for melanoma and colorectal cancer, driven by frequent V600E mutations promoting tumor growth [19,20]. BRAF inhibitors target mutated BRAF, halting its aberrant function and suppressing cancer cell growth [21]. Combining BRAF inhibitors with EGFR-targeted drugs shows promise in enhancing patient outcomes and tackling resistance [22,23]. Simultaneous EGFR and BRAF inhibition exhibits potential for increased efficacy and overcoming resistance [24,25]. Targeting EGFR and BRAF concurrently in preclinical studies shows synergistic antitumor effects [26,27].

**Figure 1:**
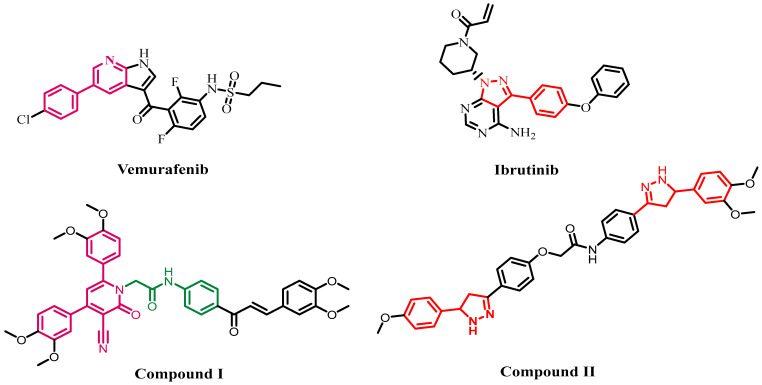
Structures of vemurafenib, Ibrutinib, compound **I**, and **II**.

Pyridine derivatives are organic compounds containing a pyridine ring, which is a six-membered aromatic ring with one nitrogen atom [28]. Pyridine derivatives hold medicinal value and versatile synthetic roles due to their heterocyclic nature [29]. Cyanopyridine derivatives have shown potential as antimicrobial agents, antibiotics [30], analgesics [31], and anticancer agents [32,33]. The anticancer activities of these compounds have attracted significant interest due to their potential to target various biological entities such as tubulin [34], HDAC [35], and PIM-1 Kinase [36,37]. Vemurafenib (Figure 1), marketed as Zelboraf^®^, is an FDA-approved pyridine derivative designed to treat advanced melanoma. It is a selective inhibitor of the V600E-mutated BRAF kinase [38,39,40].

In a recent publication [41], we described the antiproliferative activity of a novel series of cyanopyridine compounds as dual EGFR/BRAF^V600E^ inhibitors. Compound I (Figure 1), in particular, was discovered to be the most active derivative, with antiproliferative action and the ability to inhibit both EGFR and BRAF^V600E^. Compound I exhibits a noteworthy IC_50_ value (0.80 µM) against Panc-1 cancer, surpassing doxorubicin (1.00 µM). Compound **I** displays comparable EGFR and BRAF^V600E^ inhibition (IC_50_: 89 nM and 65 nM), like Erlotinib (IC_50_: 60 nM and 80 nM). Compound **I**, on the other hand, induces G0/G1 cell cycle arrest while also triggering apoptosis.

Moreover, the pyrazoline scaffold is a nitrogen-containing five-membered heterocyclic structure. Pyrazoline is a dihydropyrazole derivative with a ring-based double bond and neighboring nitrogen atoms. Pyrazoline ring cyclization via Michael addition occurs with chalcones and hydrazine monohydrate under basic conditions [42,43]. Due to its ease of synthesis and notable pharmacological and biological activities, especially concerning its anticancer properties [44,45], the pyrazoline ring has emerged as a pivotal scaffold in various heterocycles and pharmacologically active compounds [46,47]. The pyrazoline scaffold has been utilized to develop several approved drugs [48]. Ibrutinib, exemplifying exceptional anticancer activity, features a fused pyrazoline ring (Figure 1). Ibrutinib, a tyrosine kinase inhibitor, treats mantle cell lymphoma and chronic lymphocytic leukemia [49]. Pyrazoline derivatives have been documented to exhibit various pharmacological activities, including anti-diabetic [50], anti-cancer [44], anticonvulsant [51], and antidepressant [52] properties. 

We recently reported the design, synthesis, and antiproliferative activity of compound **II** (Figure 1), a pyrazoline derivative, as a dual EGFR and BRAF^V600E^ inhibitor [53]. Compound II showed an IC_50_ of 1.00 µM against A-549 cancer, outperforming doxorubicin (IC_50_: 1.40 µM). Compound **II** was effective against BRAF^V600E^, with an IC_50_ value of 93 nM, whereas Erlotinib had an IC_50_ of 60 nM. Furthermore, Compound II had an IC_50_ value of 81 nM, matching that of the EGFR inhibitor erlotinib. Pyrazoline **II** shows potential as a dual EGFR/BRAF^V600E^ inhibitor with antiproliferative efficacy.

**Figure 2:**
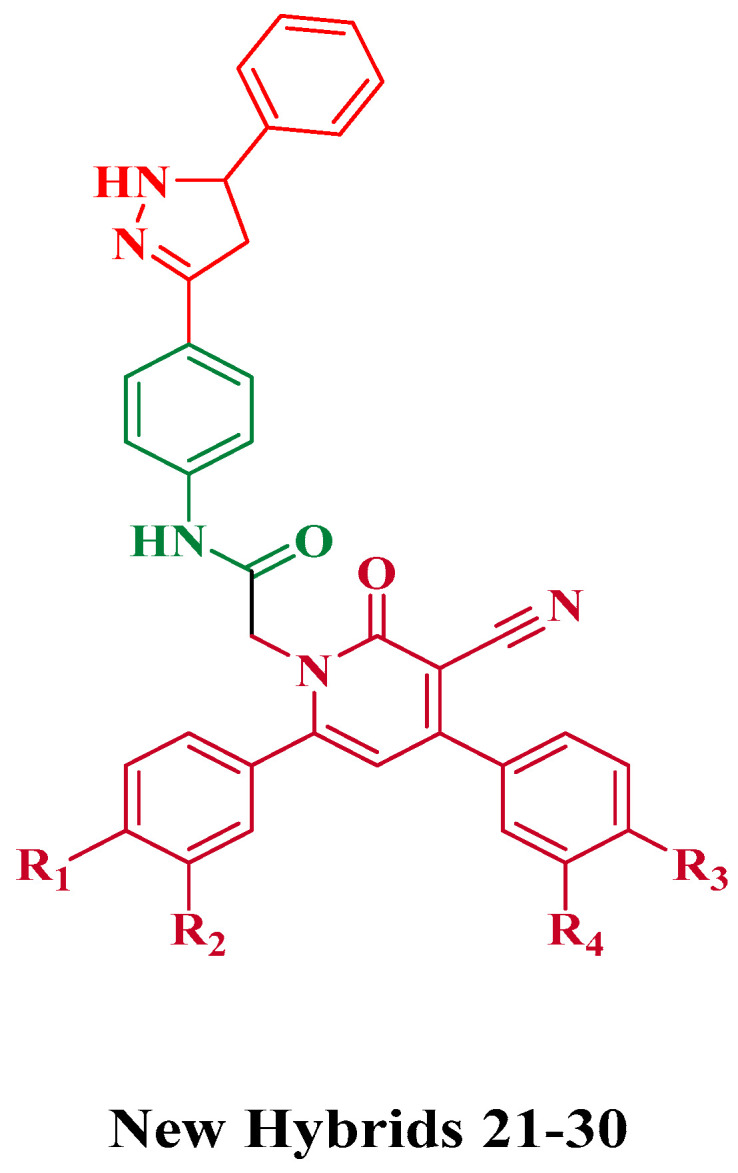
Structures of the new hybrids **21–30.**

Building on our prior anticancer research (compounds **I** and **II**), we designed and synthesized compact pyridone-2-one–pyrazoline hybrids **21–30** (Figure 2). Newly created hybrids were assessed for antiproliferative effectiveness on four human cancer cell lines. Highly active compounds were then evaluated in vitro for EGFR/BRAF^V600E^ inhibition and apoptotic effectiveness. An ADMET analysis was performed to evaluate the drug-likeness and toxicity of the novel hybrids. Molecular docking explored the binding affinities of the new pyridine/pyrazoline hybrids at EGFR and BRAF^V600E^ active sites.

## 2. Results and Discussion

### 2.1. Chemistry

**Scheme 1:**
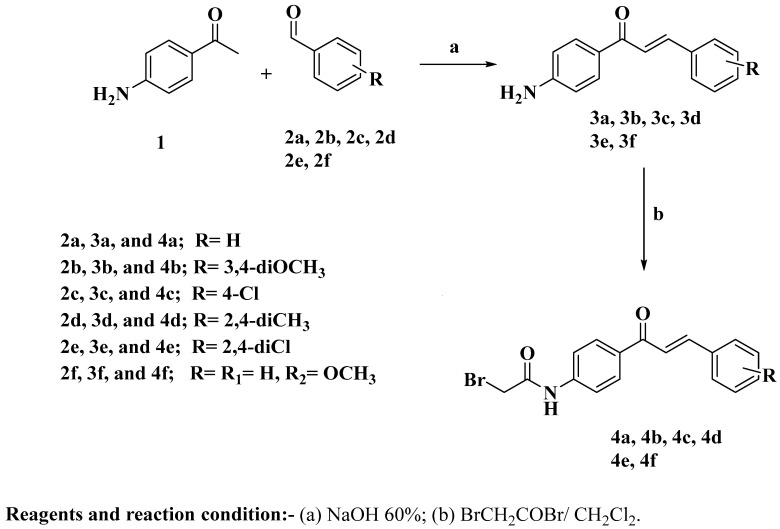
Synthesis of acetylated chalcone derivatives **4a–f.**

**Scheme 2:**
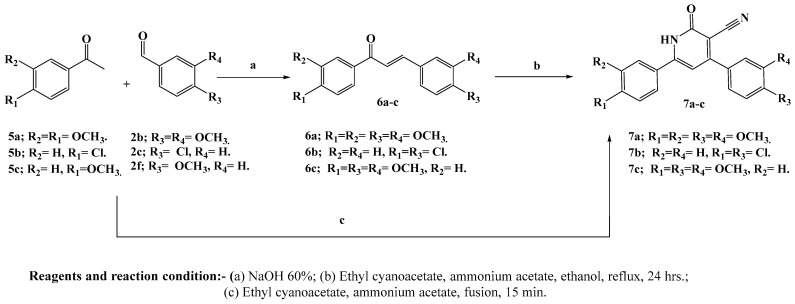
Synthesis of the intermediate compounds **7a–c.**

**Scheme 3:**
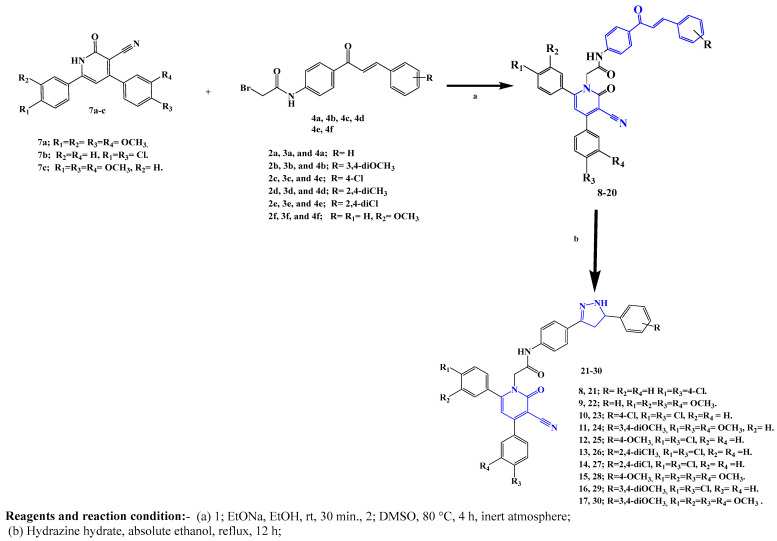
Synthesis of 3-cyanopyridone–pyrazoline hybrids **21–30.**

The synthetic procedures for the intermediates **4a–f**, **7a–c**, and the target 3-cyanopyridone–pyrazoline novel hybrids **21–30** are described in Scheme 1, Scheme 2 and Scheme 3. Shown in Scheme 1, compounds **3a–f** were synthesized through a base-catalyzed Claisen–Schmidt condensation of 4-aminoacetophenone **1** with substituted benzaldehyde derivatives **2a–f** [54]. Chalcones **3a–f** were reacted with bromoacetyl bromide in a potassium carbonate solution in dichloromethane to produce the acetylated chalcones **4a–f** [53].

The key intermediates, 3-cyano-4,6-bis(phenyl)-pyridones **7a–c**, were efficiently synthesized using a one-pot four-component reaction without any solvent. Equimolar amounts of the appropriately substituted benzaldehydes **2b, 2c**, or **2f**; the substituted acetophenones **5a, 5b,** or **5c**; ethyl cyanoacetate; and ammonium acetate were directly stirred at 110 °C for 10–15 min, resulting in the formation of the target compounds in high yields (Scheme 2) [55]. The sequential two-step reaction, which involved condensing benzaldehydes with acetophenones and then treating the resulting chalcones with ethyl cyanoacetate and excess ammonium acetate, resulted in a lower yield and a more time-consuming process. This observation highlights the advantage of the one-pot, four-component reaction used in this study, which provided a more efficient and time-saving method for synthesizing the desired compounds [32]. 

In this study, the synthesis of N-alkylated hybrids **8–20** was achieved by alkylating 3-cyanopyridones **7a–c** with acetylated chalcones **4a–f**. The reaction was performed using the sodium salt of the precursor cyanopyridones and conducted in a polar aprotic solvent, specifically dimethyl sulfoxide (DMSO), under inert conditions. This approach aimed to enhance the yield of N-alkylated hybrids **8–20**. Importantly, this method offered the convenience of not requiring chromatography to separate O-alkylated isomer by-products with low yields. Subsequently, the final 3-cyanopyridone–pyrazoline hybrids **21–30** were obtained by refluxing the N-alkylated cyanopyridones **8–20** with hydrazine monohydrate in absolute ethanol for 12 h. This synthetic pathway is depicted in Scheme 3.

**Figure 3:**
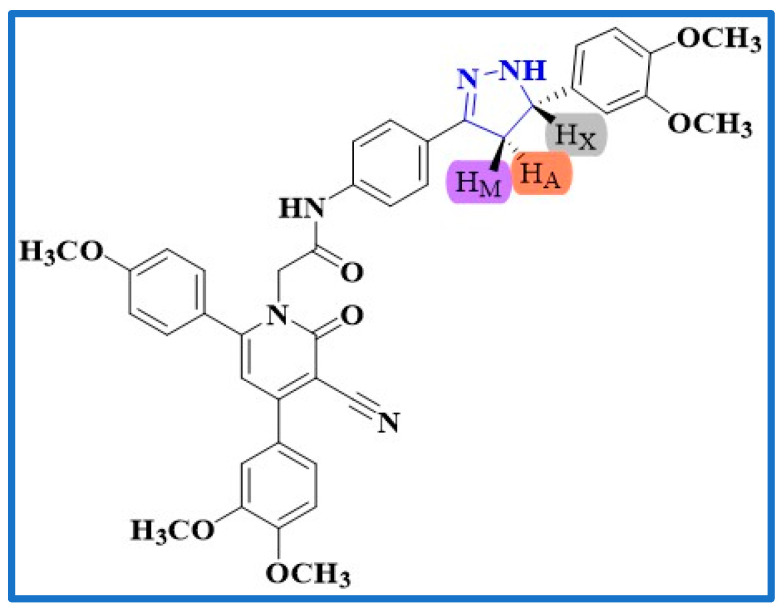
AMX pattern representation of compound **24.**

Compound **24**, a 3-cyano-4,6-(methoxyphenyl)-pyridone–pyrazoline hybrid, was subjected to ^1^H NMR spectroscopic analysis. The pyrazoline ring displayed an AMX pattern for three protons (H_A_, H_M_, and H_X_), which manifested as a doublet of a doublet at δ: 2.78 ppm (*J* values of 16.20 and 11.1 Hz) for H_A_. However, the H_M_ proton of the pyrazoline ring was split into a doublet of doublet signal at δ: 3.39 ppm (*J* values of 16.23 and 3.09 Hz), while the H_X_ protons appeared as a doublet of doublets at a higher downfield shift at δ 4.76 ppm (*J* values of 11.02 and 3.06 Hz), as illustrated in Figure 3. 

In the ^1^H NMR analysis, several signals were observed for compound **24**, confirming its structure. Two singlet signals at δ 3.74 and 3.72 ppm corresponded to the (4-phenyl-4-OCH_3_) and (6-phenyl-4-OCH_3_) groups, respectively, with three proton integrations each. The linker’s methylene protons (N-CH_2_-CO) were assigned a singlet signal at δ 5.16 ppm, indicating two proton integration. Another singlet signal at δ 7.77 ppm represented the pyridine-C5–H proton with one proton integration. The amide proton NH was identified by a singlet signal at δ 10.51 ppm.

Confirmation of the final structure was further supported by DEPTQ ^13^C NMR spectroscopy. Two signals at δ 55.88 and 55.83 ppm were assigned to the (4-phenyl-4-O**C**H_3_) and (6-phenyl-4-OCH3) groups, respectively. The methylene carbon in the pyrazoline nucleus was observed at δ 40.83 ppm, while the methine carbon of the pyrazoline ring showed a chemical shift at δ 64.00 ppm. The methylene carbon in the linker exhibited a signal at δ 66.05 ppm. Signals at δ 163.81 and 166.78 ppm indicated the presence of a pyridone carbonyl and an amide carbonyl, respectively. The remaining carbons displayed the expected chemical shifts.

### 2.2. Biological Evaluation

#### 2.2.1. Evaluation of Cell Viability 

The viability of new compounds **21–30** was examined using the human mammary gland epithelial (MCF-10A) cell line [56]. The cell viability of compounds **21–30** was assessed using the MTT assay after four days of incubation on MCF-10A cells. Table 1 shows that none of the compounds tested were cytotoxic, and all hybrids had cell viability at 50 µM of more than 88%.

#### 2.2.2. Evaluation of Antiproliferative Activity

Using Erlotinib as a control, an MTT assay was utilized to assess the antiproliferative effect of hybrids **21–30** versus four human cancer cell lines: a colon cancer (HT-29) cell line, a pancreatic cancer (Panc-1) cell line, a lung cancer (A-549) cell line, and a breast cancer (MCF-7) cell line [57]. The median inhibitory concentration (IC_50_) and GI_50_ [58] (average IC_50_) against the four cancer cell lines are shown in Table 1.

In general, the examined hybrids **21–30** revealed potent antiproliferative activity with GI_50_ values ranging from 25 nM to 42 nM versus the tested four cancer cell lines, in comparison to the standard Erlotinib, which had a GI_50_ value of 33 nM. Compounds **21**, **24**, **28**, **29**, and **30** were the most potent five derivatives, with GI_50_ values ranging from 25 nM to 30 nM, making them more potent than Erlotinib (GI_50_ = 33 nM). 

Compound **30** (R = 3,4-diOCH_3_, R_1_ = R_2_ = R_3_ = R_4_ = OCH_3_) was the most potent derivative of all newly synthesized hybrids 21–30, with a GI_50_ value of 25 nM, which is 1.3-fold more potent than the reference Erlotinib (GI_50_ = 33 nM). The 3,4-dimethoxyphenyl moiety of the pyrazole ring appears to be crucial for activity, with a drop in the number of methoxy groups related to a decrease in antiproliferative activity. For example, compound **28** (R = 4-OCH_3_, R_1_ = R_2_ = R_3_ = R_4_ = OCH_3_) ranked second in activity with a GI_50_ value of 27 nM, and compound **22** (R = H, R_1_ = R_2_ = R_3_ = R_4_ = OCH_3_), with a GI_50_ value of 33 nM, was 1.3-fold less potent than compound **30**, demonstrating the importance of the methoxy group number on the phenyl moiety at the pyrazole fifth position. Activity increased in the order 3,4-diOCH_3_ > 4-OCH_3_ > H.

Another important factor influencing the antiproliferative activity of the novel hybrids is the type of substituents on both 4, 6-diphenyl moieties of the pyridone ring. Compounds **24** (R = 3,4-diOCH_3_, R_1_ = R_3_ = R_4_ = OCH_3_, R_2_ = H) and **29** (R = 3,4-diOCH_3_, R_1_ = R_3_ = Cl, R_2_ = R_4_ = H) revealed GI_50_ values of 29 nM and 30 nM, respectively, being less potent than compound **30** but still more potent than the reference Erlotinib (GI_50_ = 33 nM).

Moreover, compound **23** (R = 4-Cl, R_1_ = R_3_ = Cl, R_2_ = R_4_ = H) revealed a GI_50_ value of 32 nM, which was less powerful than compound **29** (R = 3,4-diOCH_3_, R_1_ = R_3_ = Cl, R_2_ = R_4_ = H) (GI_50_ = 30 nM). Moreover, compounds **25** (R = 4-OCH_3_, R_1_ = R_3_ = Cl, R_2_ = R_4_ = H), **26** (R = 2,4-diCH_3_, R_1_ = R_3_ = Cl, R_2_ = R_4_ = H), and **27** (R = 2,4-diCl, R_1_ = R_3_ = Cl, R_2_ = R_4_ = H) were the least potent derivatives with GI_50_ values of 42 nM, 37 nM, and 38 nM, respectively, being less potent than compound **29**, providing more support for the importance of the dimethoxy groups of the phenyl moiety at the pyrazole fifth position.

#### 2.2.3. Evaluation of EGFR Inhibitory Activity

The five most effective antiproliferative compounds (**21**, **24**, and **28–30**) were evaluated for inhibition of EGFR as a possible target for their antiproliferative action [59]. Table 2 displays the results as IC_50_ values versus Erlotinib as a reference drug.

The results showed that the investigated hybrids **21**, **24**, and **28–30** had significant EGFR inhibitory effects, with IC_50_ values ranging from 68 nM to 75 nM, outperforming the reference Erlotinib (IC_50_ = 80 nM). Moreover, the results of an EGFR inhibitory assay were consistent with those of the antiproliferative assay, in which the most potent antiproliferative derivatives, compounds **28** (R = 4-OCH_3_, R_1_ = R_2_ = R_3_ = R_4_ = OCH_3_) and **30** (R = 3,4-diOCH_3_, R_1_ = R_2_ = R_3_ = R_4_ = OCH_3_), were the most potent EGFR inhibitors, with IC_50_ values of 70 ± 5 nM and 68 ± 5 nM, respectively, being 1.2-fold more potent than the reference Erlotinib (IC_50_ = 80 ± 5). These findings revealed that the studied compounds **21**, **24**, and **28–30** had significant EGFR inhibitory action and are potential antiproliferative agents.

#### 2.2.4. Evaluation of BRAF^V600E^ Inhibitory Activity

Hybrids **21**, **24**, and **28–30** were studied further as potential BRAF^V600E^ inhibitors. Table 2 shows the IC_50_ values compared to Erlotinib, employed as a control [60]. According to Table 2, the examined hybrids displayed a promising BRAF^V600E^ suppressive activity, with IC_50_ values ranging from 65 to 80 nM. In all cases, the examined derivatives were less potent than Erlotinib (IC_50_ = 60 nM). Compounds **28** and **30**, the most potent derivatives in the antiproliferative and EGFR suppressive assays, were likewise the most effective derivatives as anti-BRAF^V600E^, with IC_50_ values of 65 ± 5 nM and 69 ± 6 nM, respectively. These data indicate that compounds **28** and **30** exhibit substantial antiproliferative action as dual EGFR/BRAF^V600E^ inhibitors, hinting that additional structural modifications may be necessary to develop a more potent lead molecule for future development.

#### 2.2.5. Valuation of Apoptotic Activity

One approach to treating cancer is regulating or terminating the uncontrolled multiplication of cancer cells. Using the cell’s natural dying process is an extremely effective method. Apoptosis evasion is a characteristic of cancer and is not specific to the etiology or type of cancer; thus, targeting apoptosis is beneficial for many types of cancer. Many anticancer drugs target various stages in both the intrinsic and extrinsic pathways [61,62,63]. Compounds **21**, **28**, and **30**, the most effective derivatives in all in vitro studies, were examined for their ability to trigger the apoptosis cascade and reveal their proapoptotic potential.

#### 2.2.6. Caspase 3 Activation Assay

Caspases are essential for the induction and maintenance of apoptosis. Caspase-3 is an important caspase that cleaves several cell proteins, causing apoptosis [64,65]. Compounds **21**, **28**, and **30** were investigated as caspase-3 activators against the human epithelial cancer cell line (A-594) [66], and the results are shown in Table 3.

Compounds **21**, **28**, and **30** demonstrated promising caspase-3 protein overexpression levels of 530 ± 5, 590 ± 5, and 710 ± 6 pg/mL, respectively. Compared to untreated control cells, they increased the protein caspase-3 in the A-594 cancer cell line by about 8-, 9-, and 11-fold. Compounds **21**, **28**, and **30** were more active than standard staurosporine, which had a caspase-3 overexpression level of 465 ± 4 pg/mL. Compound **30**, the most effective antiproliferative agent, was once again the most active caspase-3 activator. These findings indicate the apoptotic potential of the studied compounds, which could explain their antiproliferative effect.

#### 2.2.7. Caspase-8, Bax, and Bcl-2 Levels Assay

Compounds **28** and **30** were studied further for their influence on caspase-8, Bax, and antiapoptotic Bacl-2 levels against the A-594 cancel cell line using staurosporine as a control. Results are shown in Table 3. Caspase-8 overexpression was found to be highest in compound **30** (2.50 ng/mL), followed by compound **28** (2.25 ng/mL) and the reference staurosporine (1.85 ng/mL). Compared to the untreated control cell, compounds **28** and **30** elevated caspase-8 levels by 25- and 28-fold, respectively.

Compared to untreated A-594 cancer cells, compounds **28** and **30** induced Bax 36- and 38-fold (325 pg/mL and 345 pg/mL, respectively), more than staurosporine (288 pg/mL, a 32-fold induction). Finally, compared to staurosporine, compounds **28** and **30** triggered equipotent down-regulation of anti-apoptotic Bcl-2 protein levels in the A-594 cell line. These findings imply that compounds **28** and **30** serve as caspase-3 and -8 and Bax activators and down-regulators of the anti-apoptotic Bcl-2, and can be categorized as apoptotic inducers.

### 2.3. In Silico Studies

#### 2.3.1. Docking Study

Utilizing in silico molecular docking simulation models, we tested compounds **28** and **30** against the EGFR and BRAF^V600E^ [67] proteins. The objective was to determine the binding affinity and elucidate the inhibition mechanisms of the most potent compounds (**28, 30**) with potential cellular targets within this class. The results yielded a promising outlook throughout the assessment of cellular macromolecules employed in this investigation. Employing the Discovery Studio program, we conducted molecular docking studies involving crystal structures to investigate the binding modes of the EGFR (PDB ID: 1M17) [68] and BRAF^V600E^ (BRAFm; PDB ID: 3OG7) [69]. 

The docking model involving the co-crystallized ligand (Erlotinib) positioned within the EGFR active site, which exhibited a docking score (S) of -7.7 kcal/mol and an RMSD of 1.33 Å upon re-docking within the same site. With this established and dependable docking model, we investigated the potential binding interactions of compounds **28** and **30** within the EGFR active site.

**Figure 4:**
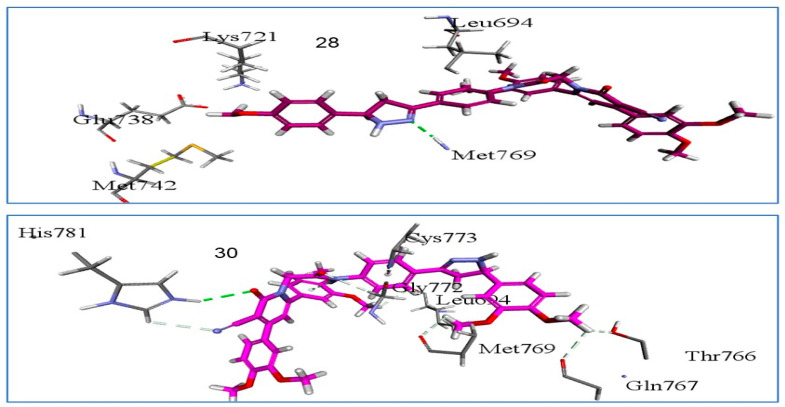
Three-dimensional design of compounds **28** and **30** within the active site of EGFR (PDB ID: 1M17) showing the binding pocket molecular surface occupied completely by novel hybrids.

Through an analysis of the optimal docking pose, observed in Figure 4, for hybrids **28** and **30**, a noteworthy pattern emerged, wherein the hybrids established a consistent hydrogen bond connection with the critical EGFR amino acid Met769. This hydrogen bond interaction was also observed between the Erlotinib pyrimidine nitrogen and Met769, as illustrated in Figure 4. Furthermore, compound **30** displayed an additional set of hydrogen bonding interactions with HIS 781, LEU 694, GLY 772, and GLN 767, in conjunction with pi–sigma interactions involving the amino acid residue CYS 773 (as depicted in Figure 4). These findings harmonize with the outcomes of the in vitro EGFR inhibition assay. 

**Figure 5:**
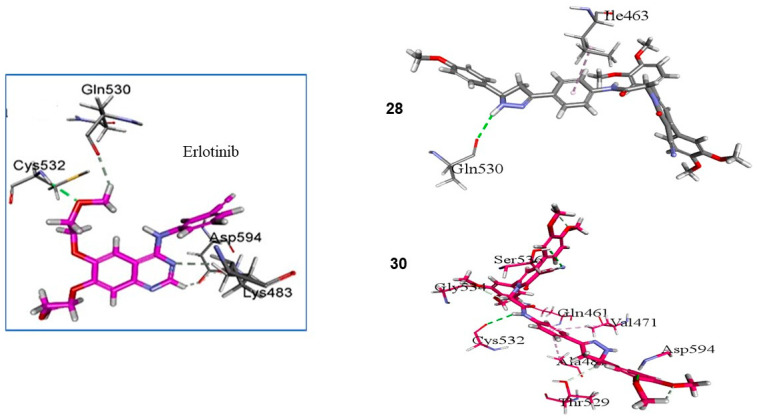
Three-dimensional binding interaction diagrams of **28**, **30,** and **Erlotinib** within the active site of BRAF^V600E^ (BRAFm; PDB ID: 3OG7).

In this context, the most potent hybrids, **28** and **30**, underwent a subsequent docking procedure within the active site of BRAFm. Upon inspecting the 2D representation of the molecular docking poses for compounds **28** and **30**, as illustrated in Figure 5, a favorable alignment within the BRAFm active site was discernible, accompanied by an array of bonding interactions.

Furthermore, upon probing the interactions between compound **28** and the BRAF active site, a typical hydrogen bond interaction with GLN 530, reminiscent of Erlotinib, was observed(Figure 5). Conversely, compound **30** exhibited a significant array of vital interactions with CYS 532, SER 536, and ASP 594. Additionally, the two methoxy groups of the pyridone moiety formed supplementary interactions with GLY 534 and GLN 461. Notably, these findings harmonize coherently with the in vitro BRAF inhibition assay outcomes.

#### 2.3.2. ADMET Studies

**Figure 6:**
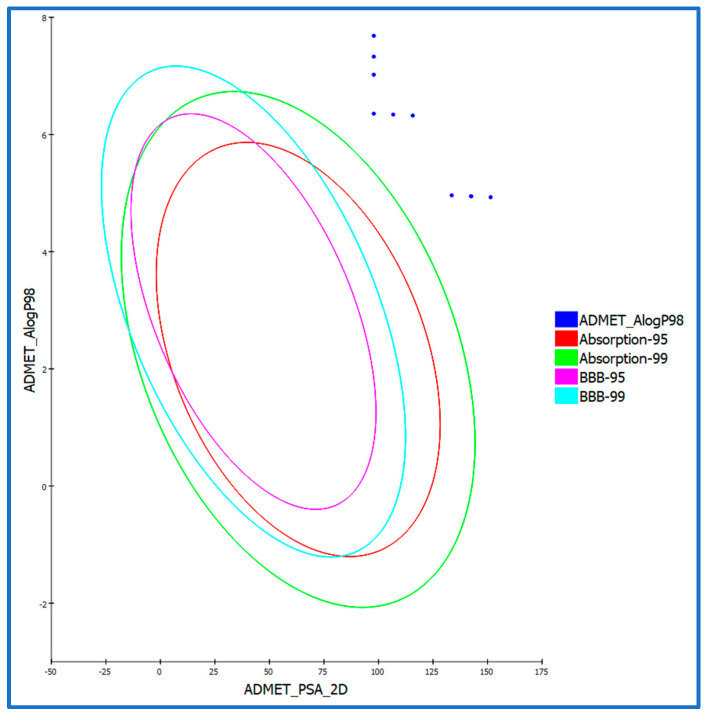
The predicted ADMET study of 3-cyanopyridone–pyrazoline hybrids **21–30**.

Given the compelling in vitro and silico docking outcomes, we undertook supplementary ADMET studies for the synthesized compounds. This decision was driven by the desire to further enhance our understanding of these pivotal activities [70]. In the ADMET investigations, Erlotinib served as the established reference compound. Utilizing Discovery Studio 4.0, we predicted the ADMET descriptors for all the compounds. The anticipated descriptors are provided in Table 4 and Figure 6. Each hybrid compound exhibited a modest predicted level of intestinal absorption (absorption level = 2), positioning them as promising candidates for localized treatment of gastrointestinal tumors or potential candidates for intravenous administration. Most of these novel hybrids demonstrated a low aqueous solubility (ADME aqueous solubility level = 1), indicating a dependency on pH for solubility. The solubility improved as the pH decreases and ionization occurs. Additionally, the creation of hydrochloride salts presents an auxiliary avenue to enhance the solubility of these hybrids.

In the ADMET assessment, all the newly synthesized hybrids were situated at a blood–brain barrier (BBB) level of 4, effectively preventing their penetration across the BBB. Notably, drug bioavailability was linked with the fundamental property of the 2D polar surface area (ADMET 2D PSA). Employing the calculated 2D polar surface area (PSA 2D) and atom-based Log P98 (A log P98) properties, the outcomes were visualized in the form of a 2D ADMET plot (Figure 6). Notably, molecules possessing a PSA of >145 generally exhibit low bioavailability and passive absorption characteristics [71]. Employing a 2D chemical structure as input, the model for cytochrome P450 2D6 (CYP2D6) predicts the inhibition potential of the CYP2D6 enzyme. The liver enzyme CYP2D6 plays a pivotal role in the metabolism of numerous substrates, contributing significantly to most drug–drug interaction scenarios [72]. Consequently, an experiment to assess CYP2D6 inhibition is imperative within the regulatory protocols employed during drug discovery and development [73]. Every assessed hybrid compound was predicted to exhibit non-inhibitory behavior towards CYP2D6. Consequently, the likelihood of inducing liver dysfunction after administering these hybrids is minimal.

The plasma protein binding model aids in determining whether a substance will exhibit strong binding (>90% bound) to blood carrier proteins. A notable binding to plasma proteins (>90%) was anticipated for most hybrid compounds, as outlined in Table 4. 

#### 2.3.3. In Silico Toxicity Predictions

Toxicity prediction was performed for the synthesized compounds using the constructed and validated models within the Discovery Studio software [BIOVIA Corp V16.1.0.15350] [74]. The rodent carcinogenicity test conducted by the FDA assesses the potential of a chemical structure to induce cancer in rats. The rat maximum tolerated dose (MTD) prediction estimates the maximum dose at which a chemical substance can be administered to rats without causing adverse effects [75]. During toxicity assessments for a chemical compound, the rat oral LD_50_ prediction is employed to anticipate the rat acute median lethal dose (LD_50_) following oral administration [76]. Within the framework of the Draize test, ocular irritancy analysis is employed to ascertain the potential of a specific compound to induce ocular irritation and to gauge the extent of the irritation severity [77]. In rabbit-based assessments, skin irritancy investigations determine the likelihood of a substance causing skin irritation and the degree of severity it might induce. Most compounds exhibited low toxicity and demonstrated minimal adverse effects according to in silico assessments, as presented in Table 5.

Furthermore, all the tested hybrids were predicted to possess non-carcinogenic properties, as initially determined by the FDA rodent carcinogenicity assessment. The evaluated compounds displayed rat oral LD_50_ values of 0.932 to 14.088 mg/kg body weight/day, similar to the Erlotinib value (0.662 mg/kg body weight/day).

Moreover, the predictive models indicated that all hybrids are expected to induce no or mild irritation in the cases of skin and ocular irritancy.

To summarize, the undertaken ADMET investigations within this study give vital insights into the newly developed hybrids’ potential effectiveness, safety, and pharmacokinetic behavior. The knowledge gleaned from these assessments holds paramount significance in steering the drug discovery and development trajectory. This wealth of information aids in pinpointing promising drug candidates worthy of further evaluation and advancement in the testing and developmental phases.

### 2.4. Structure–Activity Relationship (SAR) 

Based on the observed results, the structure–activity relationship of our novel pyridone/pyrazoline hybrids (**21**–**30**) is as follows: 
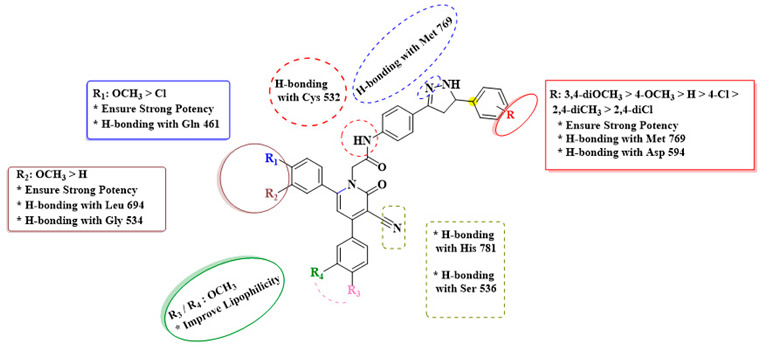


In summary, the presence and arrangement of substituents on the pyrazole and pyridone moieties appear to influence the potency of the new pyridone–pyrazoline hybrids. The involvement of specific functional groups, such as dimethoxy groups, and their positions are critical in defining the antiproliferative efficacy of these molecules.

## 3. Materials and Methods

### 3.1. Chemistry

General Details: See Supplementary Materials File.

Acetylated chalcones **4a**-**f,** 3-cyanopyridones **7a**-**c**, and 3-cyanopyridones-chalcones **8**–**20** were synthesized using previously reported procedures [55].

#### General Procedure for the Synthesis of Compounds **21**–**30**

In a 50 mL round-bottom flask, a mixture of the appropriate 3-cyanopyridone-chalcone **8**–**20** (1.32 mmol) and hydrazine monohydrate (0.2 mL, 8.92 mmol) in 30 mL of absolute ethanol was heated under reflux for 12 h. The resulting mixture was cooled to room temperature and filtered through a Buchner funnel to collect the solid. The desired solid was washed with cold ethanol to remove impurities, affording the target 3-cyanopyridone/pyrazoline novel hybrids **21**–**30**.

*2-(3-Cyano-4,6-bis(4-chlorophenyl)-2-oxopyridin-1(2H)-yl)-N-(4-(5-phenyl-4,5-dihydro-1H-pyrazol-3-yl)phenyl)acetamide (***21***)*. White crystals (0.71 g, 82% yield); m.p 273–274 °C; ^1^H NMR (500 MHz, DMSO-*d_6_*) δ (ppm): 10.30 (1H, s, O=C-NH), 8.30 (2H, d, *J* = 8.50 Hz, Ar-H), 8.21 (1H, s, pyrazoline NH), 7.77 (3H, m, Ar-H), 7.73–7.69 (5H, m, Ar-H), 7.64 (2H, d, *J* = 8.50 Hz, Ar-H), 7.60–7.55 (5H, m, Ar-H), 7.36 (1H, s, Ar-H), 5.42 (2H, s, NCH_2_), 4.85–4.83 (1H, m, pyrazoline H), 3.44 (1H, dd, *J* = 16.23 and 3.09 Hz, pyrazoline H), 2.84 (1H, dd, *J* = 16.20 and 11.01 Hz, pyrazoline H); ^13^C NMR (125 MHz, DMSO-*d_6_*) δ (ppm): 167.17, 164.82, 157.12, 156.35, 154.68, 153.64, 147.69, 138.06, 136.75, 136.21, 136.03, 135.61, 131.27, 131.00, 130.88, 130.53, 129.77, 129.52, 129.42, 129.25, 129.14, 128.87, 127.11, 112.77, 109.67, 92.86, 66.33, 64.10, 40.82; Anal. Calcd. For C_35_H_25_Cl_2_N_5_O_2_ (618.52): C, 67.97; H, 4.07; N, 11.32. Found: C, 67.66; H, 4.11; N, 11.45.
*2-(3-Cyano-4,6-bis(3,4-dimethoxyphenyl)-2-oxopyridin-1(2H)-yl)-N-(4-(5-phenyl-4,5-dihydro-1H-pyrazol-3-yl)phenyl)acetamide (***22***)*. White crystals (0.81 g, 86% yield); m.p 262–263 °C; ^1^H NMR (500 MHz, DMSO-*d*_6_) δ (ppm): 10.50 (1H, s, O=C-NH), 7.85–7.81 (2H, m, pyrazoline NH + Ar-H), 7.70 (1H, s, Ar-H), 7.62 (2H, d, *J* = 8.50 Hz, Ar-H), 7.57 (2H, d, *J* = 8.50 Hz, Ar-H), 7.49 (1H, s, Ar-H), 7.33–7.39 (6H, m, Ar-H), 7.26 (1H, s, Ar-H), 7.18 (1H, d, *J* = 9.00 Hz, Ar-H), 6.96 (1H, d, *J* = 9.00 Hz, Ar-H), 5.19 (2H, s, NCH_2_), 4.81 (1H, dd, *J* = 11.02 and 3.06 Hz, pyrazoline H), 3.88 (3H, s, OCH_3_), 3.86 (3H, s, OCH_3_), 3.79 (3H, s, OCH_3_), 3.63 (3H, s, OCH_3_), 3.43 (1H, dd, *J* = 16.23 and 3.09 Hz, pyrazoline H), 2.80 (1H, dd, *J* = 16.20 and 11.01 Hz, pyrazoline H); ^13^C NMR (125 MHz, DMSO-*d*_6_) δ (ppm): 166.79, 163.77, 157.10, 156.56, 151.61, 150.87, 149.32, 149.18, 148.86, 143.54, 139.03, 129.43, 128.98, 128.86, 128.56, 127.58, 127.10, 126.53, 121.99, 121.38, 119.52, 116.30, 113.73, 112.75, 112.26, 111.98, 110.70, 91.30, 65.97, 64.10, 56.20, 56.18, 56.08, 55.82, 41.14. Anal. Calcd. For C_39_H_35_N_5_O_6_ (669.74): C, 69.94; H, 5.27; N, 10.46. Found: C, 70.03; H, 5.16; N, 10.39.
*2-(3-Cyano-4,6-bis(4-chlorophenyl)-2-oxopyridin-1(2H)-yl)-N-(4-(5-(4-chlorophenyl)-4,5-dihydro-1H-pyrazol-3-yl)phenyl)acetamide (***23***)***.** White powder (0.81 g, 89% yield); m.p 286–287 °C; ^1^H NMR (500 MHz, DMSO-*d*_6_) δ (ppm): 10.56 (1H, s, O=C-NH), 8.24 (2H, d, *J* = 8.50 Hz, Ar-H), 7.91 (1H, s, pyrazoline NH), 7.81 (2H, d, *J* = 8.50 Hz, Ar-H), 7.70–7.60 (8H, m, Ar-H), 7.85–7.81 (5 H, m, Ar-H), 5.20 (2H, s, NCH_2_), 4.81–4.85 (1H, m, pyrazoline H), 3.45 (1H, dd, *J* = 16.23 and 3.09 Hz, pyrazoline H), 2.79 (1H, dd, *J* = 11.23 and 3.03 Hz, pyrazoline H); ^13^C NMR (125 MHz, DMSO-*d*_6_) δ (ppm): 166.57, 163.68, 156.20, 155.94, 148.99, 142.59, 138.93, 136.23, 135.73, 135.51, 134.85, 132.03, 131.09, 129.72, 129.44, 129.31, 129.01, 128.93, 128.80, 126.61, 119.71, 115.45, 114.80, 93.08, 66.26, 63.33, 41.10; Anal. Calcd. For C_35_H_24_Cl_3_N_5_O_2_ (652.96): C, 64.38; H, 3.70; N, 10.73. Found: C, 64.46; H, 3.82; N, 10.59.
*2-(3-Cyano-4-(3,4-dimethoxyphenyl)-6-(4-methoxyphenyl)-2-oxopyridin-1(2H)-yl)-N-(4-(5-(3,4-dimethoxyphenyl)-4,5-dihydro-1H-pyrazol-3-yl)phenyl)acetamide (***24***)*. White crystals (0.86 g, 88% yield); m.p 244–245 °C; ^1^H NMR (500 MHz, DMSO-*d*_6_) δ (ppm): 10.50 (1H, s, O=C-NH), 8.16 (2H, d, *J* = 8.5 Hz, Ar-H), 7.77 (1H, s, pyrazoline NH), 7.58–7.66 (4H, m, Ar-H), 7.34–7.41 (3H, m, Ar-H + pyridine-C5–H), 7.16 (1H, d, *J* = 8.5 Hz, Ar-H), 6.88–6.99 (5H, m, Ar-H), 5.16 (2H, s, NCH_2_), 4.78 (1H, dd, *J* = 11.02 and 3.06 Hz, pyrazoline H), 3.87 (3H, s, OCH_3_), 3.86 (3H, s, OCH_3_), 3.79 (3H, s, OCH_3_), 3.74 (3H, s, OCH_3_), 3.72 (3H, s, OCH_3_), 3.39 (1H, dd, *J* = 16.23 and 3.09 Hz, pyrazoline H), 2.81 (1H, dd, *J* = 16.20 and 11.01 Hz, pyrazoline H); ^13^C NMR (125 MHz, DMSO-*d*_6_) δ (ppm): 166.78, 163.81, 161.85, 157.01, 156.62, 150.90, 149.20, 149.13, 148.41, 138.92, 135.77, 129.69, 129.28, 129.10, 128.51, 126.51, 121.99, 119.65, 119.09, 116.27, 114.61, 113.49, 112.68, 112.23, 112.16, 112.12, 110.84, 91.22, 66.05, 64.00, 56.18, 56.16, 56.03, 55.88, 55.83, 40.83. Anal. Calcd. For C_40_H_37_N_5_O_7_ (699.76): C, 68.66; H, 5.33; N, 10.01. Found: C, 68.58; H, 5.21; N, 10.12.
*2-(3-Cyano-4,6-bis(4-chlorophenyl)-2-oxopyridin-1(2H)-yl)-N-(4-(5-(4-methoxyphenyl)-4,5-dihydro-1H-pyrazol-3-yl)phenyl)acetamide (***25***)*. White powder (0.83 g, 92% yield); m.p 256–257 °C; ^1^H NMR (500 MHz, DMSO-*d*_6_) δ (ppm): 10.53 (1H, s, O=C-NH), 8.22 (2H, d, *J* = 8.50 Hz, Ar-H), 7.89 (1H, s, pyrazoline NH), 7.80 (2H, d, *J* = 8.50 Hz, Ar-H), 7.58–7.69 (6H, m, Ar-H), 7.46–7.40 (3H, m, Ar-H), 7.28 (2H, d, *J* = 8.50 Hz, Ar-H), 6.89 (2H, d, *J* = 8.50 Hz, Ar-H), 5.20 (2H, s, NCH_2_), 4.73–4.78 (1H, m, pyrazoline H), 3.79-385 (1H, m, pyrazoline H), 3.72 (3H, s, OCH_3_), 2.77 (1H, dd, *J* = 16.20 and 11.01 Hz, pyrazoline H); ^13^C NMR (125 MHz, DMSO-*d*_6_) δ (ppm): 166.74, 163.20, 155.79, 155.38, 135.83, 135.36, 134.99, 134.32, 130.57, 129.19, 128.97, 128.82, 128.55, 127.78, 127.16, 126.49, 126.12, 125.70, 119.34, 115.00, 114.22, 113.78, 113.45, 92.59, 65.83, 63.26, 55.54, 39.85; Anal. Calcd. For C_36_H_27_Cl_2_N_5_O_3_ (648.54): C, 66.67; H, 4.20; N, 10.80. Found: C, 66.75; H, 4.36; N, 10.73.
*2-(3-Cyano-4,6-bis(4-chlorophenyl)-2-oxopyridin-1(2H)-yl)-N-(4-(5-(2,4-dimethylphenyl)-4,5-dihydro-1H-pyrazol-3-yl)phenyl)acetamide (*26*)*. White crystals (0.78 g, 86% yield); m.p 269–270 °C; ^1^H NMR (500 MHz, DMSO-*d*_6_) δ (ppm): 10.52 (1H, s, O=C-NH), 8.21 (2H, d, *J* = 8.50 Hz, Ar-H), 7.90 (1H, s, pyrazoline NH), 7.81–7.79 (3H, m, Ar-H), 7.69–7.58 (6H, Ar-H), 7.45 (2H, d, *J* = 8.50 Hz, Ar-H), 7.29 (1H, d, *J* = 8.50 Hz, Ar-H), 6.96 (2H, d, *J* = 8.50 Hz, Ar-H), 5.20 (2H, s, NCH_2_), 4.92 (1H, dd, *J* = 11.02 and 3.06 Hz, pyrazoline H), 3.44 (1H, dd, *J* = 16.23 and 3.09 Hz, pyrazoline H), 2.65 (1H, dd, *J* = 16.20 and 11.01 Hz, pyrazoline H), 2.28 (3H, s, CH_3_), 2.22 (3H, s, CH_3_); ^13^C NMR (125 MHz, DMSO-*d*_6_) δ (ppm): 166.54, 163.70, 156.21, 155.94, 148.44, 138.78, 138.54, 136.23, 136.16, 135.73, 135.53, 135.26, 134.87, 131.37, 131.11, 129.74, 129.50, 129.31, 126.92, 126.51, 126.06, 119.69, 119.63, 115.46, 114.83, 93.09, 66.27, 60.74, 40.47, 21.01, 19.45; Anal. Calcd. For C_37_H_29_Cl_2_N_5_O_2_ (646.57): C, 68.73; H, 4.52; N, 10.83. Found: C, 68.88; H, 4.46; N, 10.74.
*2-(3-Cyano-4,6-bis(4-chlorophenyl)-2-oxopyridin-1(2H)-yl)-N-(4-(5-(2,4-dichlorophenyl)-4,5-dihydro-1H-pyrazol-3-yl)phenyl)acetamide (***27***)*. White powder (0.78 g, 91% yield); m.p 291–292 °C; ^1^H NMR (500 MHz, DMSO-*d*_6_) δ (ppm): 10.52 (1H, s, O=C-NH), 8.22 (2H, d, *J* = 8.50 Hz, Ar-H), 7.91 (1H, s, pyrazoline NH), 7.82 (2H, d, *J* = 8.50 Hz, Ar-H), 7.70–7.57 (9H, m, Ar-H), 7.47–7.43 (3H, m, Ar-H), 5.20 (2H, s, NCH_2_), 5.05 (1H, dd, *J* = 11.02 and 3.06 Hz, pyrazoline H), 3.58 (1H, dd, *J* = 16.23 and 3.09 Hz, pyrazoline H), 2.73 (1H, dd, *J* = 16.20 and 11.01 Hz, pyrazoline H); ^13^C NMR (125 MHz, DMSO-*d*_6_) δ (ppm): 166.77, 163.21, 155.72, 155.44, 139.68, 138.59, 135.75, 135.28, 135.02, 134.35, 132.71, 132.31, 131.64, 130.62, 130.08, 129.24, 128.96, 128.82, 128.20, 127.75, 127.57, 126.24, 119.21, 114.93, 114.33, 92.61, 65.79, 59.88, 40.15; Anal. Calcd. For C_35_H_23_Cl_4_N_5_O_2_ (687.40): C, 61.16; H, 3.37; N, 10.19. Found: C, 61.23; H, 3.43; N, 10.25.
*2-(3-Cyano-4,6-bis(3,4-dimethoxyphenyl)-2-oxopyridin-1(2H)-yl)-N-(4-(5-(4-methoxyphenyl)-4,5-dihydro-1H-pyrazol-3-yl)phenyl)acetamide (***28***)*. White powder (0.81 g, 83% yield); m.p 256–257 °C; ^1^H NMR (500 MHz, DMSO-*d*_6_) δ (ppm): 10.53 (1H, s, O=C-NH), 8.17 (2H, d, *J* = 8.5 Hz, Ar-H), 7.77 (1H, s, pyrazoline NH), 7.65 (2H, d, *J* = 8.50 Hz, Ar-H), 7.60 (2H, d, *J* = 8.50 Hz, Ar-H), 7.41 (1H, s, Ar-H), 7.36 (2H, d, *J* = 8.5 Hz, Ar-H), 7.17 (1H, s, Ar-H), 6.99–6.88 (5H, m, Ar-H), 5.16 (2H, s, NCH_2_), 4.76 (1H, dd, *J* = 11.02 and 3.06 Hz, pyrazoline H), 3.87 (3H, s, OCH_3_), 3.86 (3H, s, OCH_3_), 3.79 (3H, s, OCH_3_), 3.74 (3H, s, OCH_3_), 3.73 (3H, s, OCH_3_), 3.44 (1H, dd, *J* = 16.23 and 3.09 Hz, pyrazoline H), 2.81 (1H, dd, *J* = 16.20 and 11.01 Hz, pyrazoline H); ^13^C NMR (125 MHz, DMSO-*d*_6_) δ (ppm): 166.99, 163.36, 161.47, 156.62, 156.27, 153.09, 150.53, 148.82, 143.48, 142.79, 129.47, 129.27, 128.86, 128.62, 128.10, 127.37, 121.86, 121.62, 118.93, 115.84, 114.22, 113.18, 112.30, 111.86, 111.36, 110.05, 92.02, 90.84, 65.67, 62.13, 55.86, 55.83, 55.81, 55.78, 55.44, 38.89. Anal. Calcd. For C_40_H_37_N_5_O_7_ (699.76): C, 68.66; H, 5.33; N, 10.01. Found: C, 68.58; H, 5.26; N, 10.09.
*2-(3-Cyano-4,6-bis(4-chlorophenyl)-2-oxopyridin-1(2H)-yl)-N-(4-(5-(3,4-dimethoxyphenyl)-4,5-dihydro-1H-pyrazol-3-yl)phenyl)acetamide (***29***)*. White crystals (0.79 g, 83% yield); m.p 243–245 °C; ^1^H NMR (500 MHz, DMSO-*d*_6_) δ (ppm): 10.53 (1H, s, O=C-NH), 8.23 (2H, d, *J* = 8.50 Hz, Ar-H), 7.91 (1H, s, pyrazoline NH), 7.82 (2H, d, *J* = 8.50 Hz, Ar-H), 7.70–7.58 (7H, m, Ar-H),), 7.47–7.40 (3H, m, Ar-H), 6.99 (1H, s, Ar-H), 6.89 (1H, d, *J* = 8.50 Hz, Ar-H), 5.20 (2H, s, NCH_2_), 4.76 (1H, dd, *J* = 11.02 and 3.06 Hz, pyrazoline H), 3.74 (3H, s, OCH_3_), 3.72 (3H, s, OCH_3_), 3.37 (1H, m, pyrazoline H), 2.80 (1H, dd, *J* = 16.20 and 11.01 Hz, pyrazoline H); ^13^C NMR (125 MHz, DMSO-*d*_6_) δ (ppm): 166.09, 163.25, 155.74, 155.41, 148.73, 148.62, 147.98, 138.38, 135.76, 135.30, 135.02, 134.34, 130.60, 129.23, 128.95, 128.81, 128.55, 126.07, 119.27, 118.83, 118.64, 114.96, 114.29, 111.70, 110.41, 92.60, 65.81, 63.57, 55.56, 55.42, 40.64. Anal. Calcd. For C_37_H_29_Cl_2_N_5_O_4_ (678.57): C, 65.49; H, 4.31; N, 10.32. Found: C, 65.57; H, 4.44; N, 10.40.
*2-(3-Cyano-4,6-bis(3,4-dimethoxyphenyl)-2-oxopyridin-1(2H)-yl)-N-(4-(5-(3,4-dimethoxyphenyl)-4,5-dihydro-1H-pyrazol-3-yl)phenyl)acetamide (***30***)*. White powder (0.89 g, 87% yield); m.p 233–234 °C; ^1^H NMR (500 MHz, DMSO-*d*_6_) δ (ppm): 10.48 (1H, s, O=C-NH), 7.81–7.57 (8H, m, pyrazoline NH _+_ 7 Ar-H), 7.36 (2H, d, *J* = 8.50 Hz, Ar-H), 7.17 (1H, d, *J* = 8.50 Hz, Ar-H), 6.88–6.99 (4H, m, Ar-H), 5.19 (2H, s, NCH_2_), 4.76-4.73 (1H, m, pyrazoline H), 3.87 (3H, s, OCH_3_), 3.85 (3H, s, OCH_3_), 3.78 (3H, s, OCH_3_), 3.74 (3H, s, OCH_3_), 3.72 (3H, s, OCH_3_), 3.63 (3H, s, OCH_3_), 2.81 (1H, dd, *J* = 16.20 and 11.01 Hz, pyrazoline H); ^13^C NMR (125 MHz, DMSO-*d_6_*) δ (ppm): 166.42, 163.36, 156.67, 156.08, 151.18, 150.46, 148.91, 148.75, 148.02, 138.60, 135.33, 129.02, 128.67, 128.13, 126.09, 121.58, 120.91, 119.14, 118.96, 115.92, 113.23, 112.29, 111.97, 111.50, 110.44, 110.27, 90.86, 65.57, 63.63, 55.74, 55.71, 55.61, 55.58, 55.46, 55.39, 40.66..Anal. Calcd. For C_41_H_39_N_5_O_8_ (729.79): C, 67.48; H, 5.39; N, 9.60.

### 3.2. Biology

#### 3.2.1. Cell Viability Assay

The human mammary gland epithelial (MCF-10A) normal cell line was utilized to investigate the viability of new derivatives **21**–**30** [56]. For more information, see Supplementary Materials Files.

#### 3.2.2. Antiproliferative Assay

An MTT assay was used to assess the antiproliferative activity of **21**–**30** against four human cancer cell lines: a colon cancer (HT-29) cell line, a pancreatic cancer (Panc-1) cell line, a lung cancer (A-549) cell line, and a breast cancer (MCF-7) cell line, using Erlotinib as the control, see Supplementary Materials Files)

#### 3.2.3. EGFR Inhibitory Assay

The five most active antiproliferative compounds (**21**, **24**, and **28–30**) were evaluated for inhibition of EGFR as a possible target for their antiproliferative action [59]. See Supplementary Materials Files.

#### 3.2.4. BRAF^V600E^ Inhibitory Assay

Hybrids **21**, **24**, and **28–30** were studied further as potential BRAF^V600E^ inhibitors. **T**able 2 shows the IC_50_ values compared to Erlotinib, employed as a control [60]. See Supplementary Materials Files.

#### 3.2.5. Apoptotic Markers Assays

Compounds **21**, **28**, and **30** were investigated as caspase-3, caspase-8, Bax activators, and Bcl-2 down-regulators against the human epithelial cancer cell line (A-594) [66]. See Supplementary Materials Files.

#### 3.2.6. Docking Study

For the molecular docking study, we utilized the computational software BIOVIA I Discovery Studio 2016, provided by manufacturers located in San Diego, California. The registration addresses and copyright details are as follows: Copyright 2015, Dassault Systèmes BIOVIA Corp V16.1.0.15350. The chosen proteins underwent preparation for docking analysis via the Protein Preparation Wizard [78]. Ligands were then mapped onto a three-dimensional model and subjected to energy minimization using LigPrep. To enhance potential binding, a receptor grid was generated for the selected binding site using the Receptor Grid Generation Tool. Ultimately, the Glide tool was utilized to assess both docking scores and various binding modes for the ligands.

#### 3.2.7. In Silico ADMET Analysis

ADMET studies were performed using BIOVIA I Discovery Studio 2016 [79]. The chemical structures of all compounds were imported, and ADMET descriptors were predicted using integrated models, including Lipinski’s Rule of Five and assessments of absorption, distribution, metabolism, excretion, and toxicity. The obtained results were analyzed to ascertain the drug-likeness and safety profiles of the compounds.

## 4. Conclusions

In this study, novel 3-cyanopyridone/pyrazoline derivatives were synthesized as potential dual-targeting antiproliferative agents. Compounds **28** and **30** exhibited remarkable antiproliferative activity, surpassing Erlotinib, with notable potency against the EGFR and BRAF^V600E^ pathways. The induction of apoptosis in MCF-7 cells was linked to the upregulation of caspase-3 and an altered Bax/Bcl-2 gene ratio, thereby emphasizing the potential mechanism of compounds **28** and **30**. Molecular docking affirmed the strong inhibition of compound **30** against both EGFR and BRAF^V600E^. ADMET analysis indicated favorable safety profiles for most compounds. Further in vitro and in vivo studies, along with chemical optimizations, are warranted to enhance efficacy. Overall, this research introduces promising dual inhibitors (**28** and **30**) against EGFR/BRAF^V600E^ pathways, laying the groundwork for advanced antiproliferative agents.

## Figures and Tables

**Table 1:** Comparative analysis of antiproliferative IC_50_ values for Compounds **21–30** and Erlotinib across different cancer cell lines.

Comp.	Cell Viability %	Antiproliferative Activity IC_50_ ± SEM (nM)				
		A-549	MCF-7	Panc-1	HT-29	Average IC_50_ (GI_50_)
**21**	90	26 ± 2	30 ± 3	28 ± 2	28 ± 2	28
**22**	89	32 ± 3	35 ± 3	34 ± 3	32 ± 3	33
**23**	91	29 ± 2	34 ± 3	32± 3	32 ± 3	32
**24**	90	27 ± 2	31 ± 3	29± 2	28 ± 2	29
**25**	88	40 ± 3	45 ± 4	42 ± 4	42 ± 4	42
**26**	92	35 ± 3	40 ± 4	36 ± 3	36 ± 3	37
**27**	88	36 ± 3	42 ± 4	38 ± 3	38 ± 3	38
**28**	89	25 ± 2	30 ± 3	26 ± 2	26 ± 2	27
**29**	91	27 ± 2	31 ± 3	30 ± 3	30 ± 3	30
**30**	90	23 ± 2	28 ± 2	24 ± 2	24 ± 2	25
**Erlotinib**	ND	30 ± 3	40 ± 3	30 ± 3	30 ± 3	33

ND: Not Determined.

**Table 2:** Determination of inhibitory concentration (IC_50_) for Compounds **21, 24, 28–30** against Epidermal Growth Factor Receptor (EGFR) and BRAF^V600E^ oncogene.

Compd.	EGFR Inhibition IC_50_ ± SEM (nM)	BRAF^V600E^ Inhibition IC_50_ ± SEM (nM)
**21**	72 ± 5	73 ± 6
**24**	73 ± 5	77 ± 6
**28**	70 ± 5	69 ± 6
**29**	75 ± 5	80 ± 7
**30**	68 ± 4	65 ± 5
**Erlotinib**	80 ± 5	± 5

**Table 3:** Caspase-3 and -8, Bax, and Bcl-2 levels for compounds **21**, **28**, and **30** and **Staurosporine** on a human epithelial cancer cell line (A-594).

Compd. No.	Caspase-3		Caspase-8		Bax		Bcl-2	
	Conc (Pg/mL)	Fold Change	Conc (ng/mL)	Fold Change	Conc (Pg/mL)	Fold Change	Conc (ng/mL)	Fold Reduction
**21**	530 ± 5	8	ND	ND	ND	ND	ND	ND
**28**	595 ± 5	9	2.25	25	325	36	0.80	6
**30**	710 ± 6	11	2.50	28	345	38	0.65	8
**Staurosporine**	465 ± 4	7	1.85	21	288	32	1.00	5
**Control**	65	1	0.09	1	9	1	5.00	1

ND: Not Determined.

**Table 4:** Comprehensive prediction of the absorption, distribution, metabolism, excretion, and toxicity (ADMET) profiles of synthesized 3-cyanopyridone–pyrazoline hybrids **21–30.**

Comp. ID	PSA	PPB ^a^	Absorption Level ^b^	CYP2D6 Prediction ^c^	BBB Level ^d^	Solubility Level ^e^	AlogP98
**21**	97.832	Yes	2	No	4	1	6.356
**22**	133.553	No	2	No	4	2	4.962
**23**	97.832	Yes	3	No	4	1	7.021
**24**	142.483	No	2	No	4	3	4.945
**25**	106.763	Yes	2	No	4	1	6.34
**26**	97.832	Yes	3	No	4	1	7.329
**27**	97.832	Yes	3	No	4	1	7.685
**28**	142.483	No	2	No	4	2	4.945
**29**	115.693	Yes	2	No	4	1	6.323
**30**	151.413	Yes	3	No	4	3	4.929
**Erlotinib**	71.052	Yes	0	No	1	2	4.309

**^a^** PPB, plasma protein binding, FALSE means less than 90%, TRUE means >90%. ^b^ Absorption level, 0 = good, 1 = moderate, 2 = poor, 3 = very poor. ^c^ CYP2D6, cytochrome P2D6, TRUE = inhibitor, FALSE = non inhibitor. ^d^ BBB level, blood–brain barrier level, 0 = very high, 1 = high, 2 = medium, 3 = low, 4 = very low. ^e^ Solubility level, 1 = very low, 2 = low, 3 = good, 4 = optimal.

**Table 5:** Extensive prediction and analysis of toxicity properties for synthesized hybrid compounds (**21–30**).

Comp. ID	FDA Rodent Carcinogenicity (Mouse, Female)	Rat Maximum Tolerated Dose (Feed) ^a^	Rat Oral LD_50_ ^a^	Ocular Irritancy	Skin Irritancy
**21**	Non-Carcinogen	0.0760	1.27741	Mild	Non-Irritant
**22**	Non-Carcinogen	0.0312	8.48863	Mild	Non-Irritant
**23**	Non-Carcinogen	0.0597	1.67537	Moderate-Severe	Non-Irritant
**24**	Non-Carcinogen	0.0293	14.0883	Mild	Non-Irritant
**25**	Non-Carcinogen	0.0351	0.932102	Mild	Non-Irritant
**26**	Non-Carcinogen	0.0582	4.49508	Mild	Non-Irritant
**27**	Non-Carcinogen	0.0468	2.05463	Mild	Non-Irritant
**28**	Non-Carcinogen	0.0293	14.0883	Mild	Non-Irritant
**29**	Non-Carcinogen	0.0329	5.06156	Mild	Non-Irritant
**30**	Non-Carcinogen	0.0274	12.7428	Mild	Non-Irritant
**Erlotinib**	Non-Carcinogen	0.0828	0.662169	Mild	Non-Irritant

^a^ Unit: g/kg body weight.

## Data Availability

The data will be provided upon request.

## Supplementary Materials

The following supporting information can be downloaded at: https://www.mdpi.com/article/10.3390/molecules28186586/s1.

## Sample Availability

Samples will be available upon request form the authors.
