# Supplementary material for: Design, Synthesis, and Biological Evaluation of Novel 3-Cyanopyridone/Pyrazoline Hybrids as Potential Apoptotic Antiproliferative Agents Targeting EGFR/BRAFV600E Inhibitory Pathways
Source: Molecules. 2023 Sep 12;28(18):6586. doi: 10.3390/molecules28186586 (PMC10537368; doi:10.3390/molecules28186586)
Supplement: Supplementary file 1 [file molecules-28-06586-s001.zip › molecules-2587144-supplementary.pdf]

# Design, Synthesis, and Biological Evaluation of Novel 3-Cyanopyridone/Pyrazoline Hybrids as Potential Apoptotic Antiproliferative Agents Targeting EGFR/BRAF$^{V600E}$ Inhibitory Pathways

**Lamya H. Al-Wahaibi [1], Hesham A. Abou-Zied [2], Mohamed Hisham [2], Eman A. M. Beshr [3], Bahaa G. M. Youssif [4,*], Stefan Bräse [5,*], Alaa M. Hayallah [4,6,*] and Mohamed Abdel-Aziz [3]**

[1] Department of Chemistry, College of Sciences, Princess Nourah bint Abdulrahman University, Riyadh 11564, Saudi Arabia; lhalwahaibi@pnu.edu.sa
[2] Medicinal Chemistry Department, Faculty of Pharmacy, Deraya University, Minia 61111, Egypt; drhesham92@yahoo.com (H.A.A.-Z.); mohammedhisham90@yahoo.com (M.H.)
[3] Medicinal Chemistry Department, Faculty of Pharmacy, Minia University, Minia 61519, Egypt; emanbeshr@yahoo.com (E.A.M.B.); abulnil@hotmail.com (M.A.-A.)
[4] Pharmaceutical Organic Chemistry Department, Faculty of Pharmacy, Assiut University, Assiut 71526, Egypt
[5] Institute of Biological and Chemical Systems, IBCS-FMS, Karlsruhe Institute of Technology, 76131 Karlsruhe, Germany
[6] Pharmaceutical Organic Chemistry Department, Faculty of Pharmacy, Sphinx University, Assiut 71515, Egypt
* Correspondence: bgyoussif2@gmail.com or bahaa.youssif@pharm.aun.edu.eg (B.G.M.Y.); braese@kit.edu (S.B.); alaa_hayalah@yahoo.com (A.M.H.); Tel.: +20-1098294419 (B.G.M.Y.); +20-1283410110 (A.M.H.)

# Supporting information

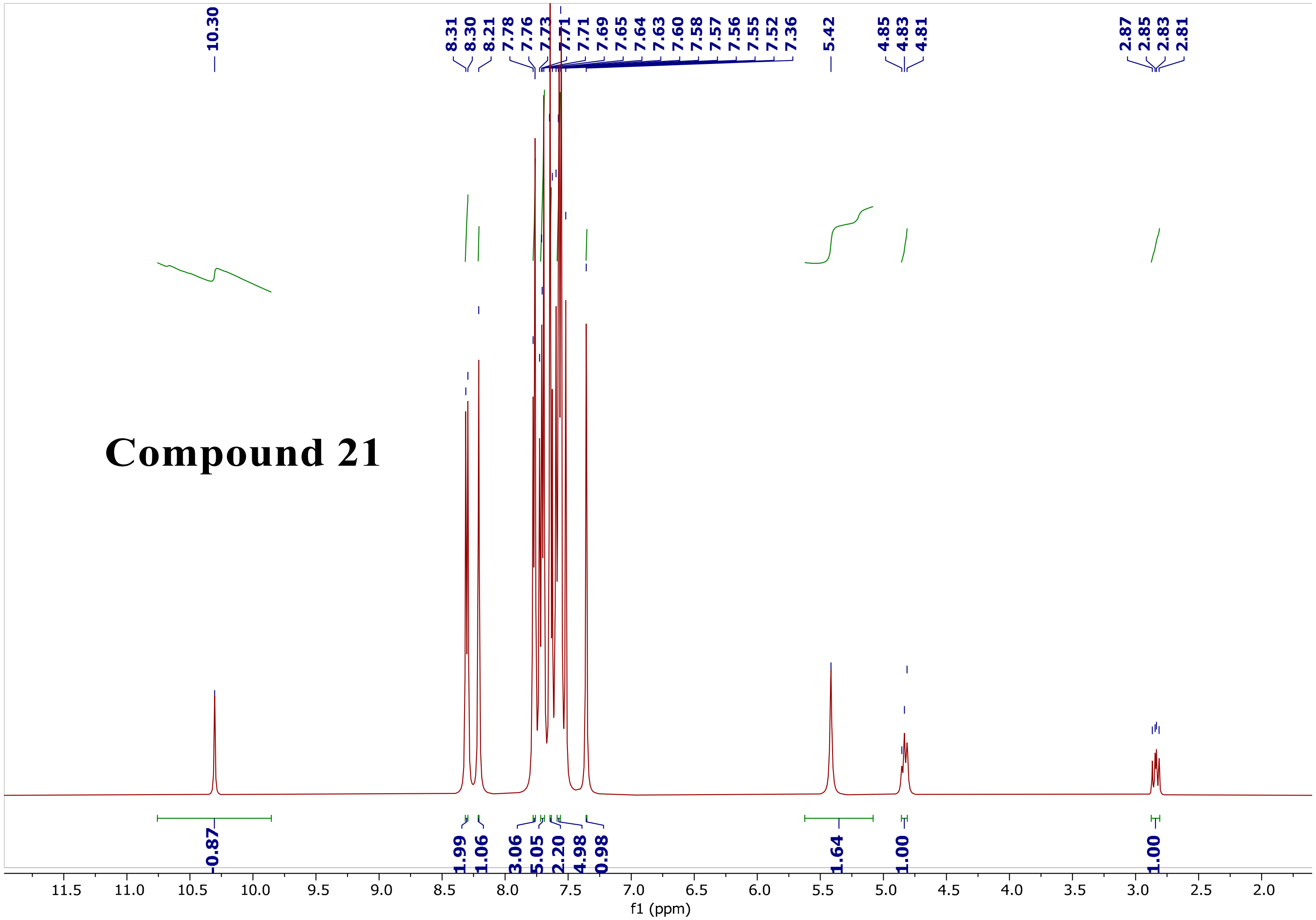


**Figure S1.** $^{1}$H NMR spectrum for compound **21** in DMSO-*d*$_6$ (500 MHz).

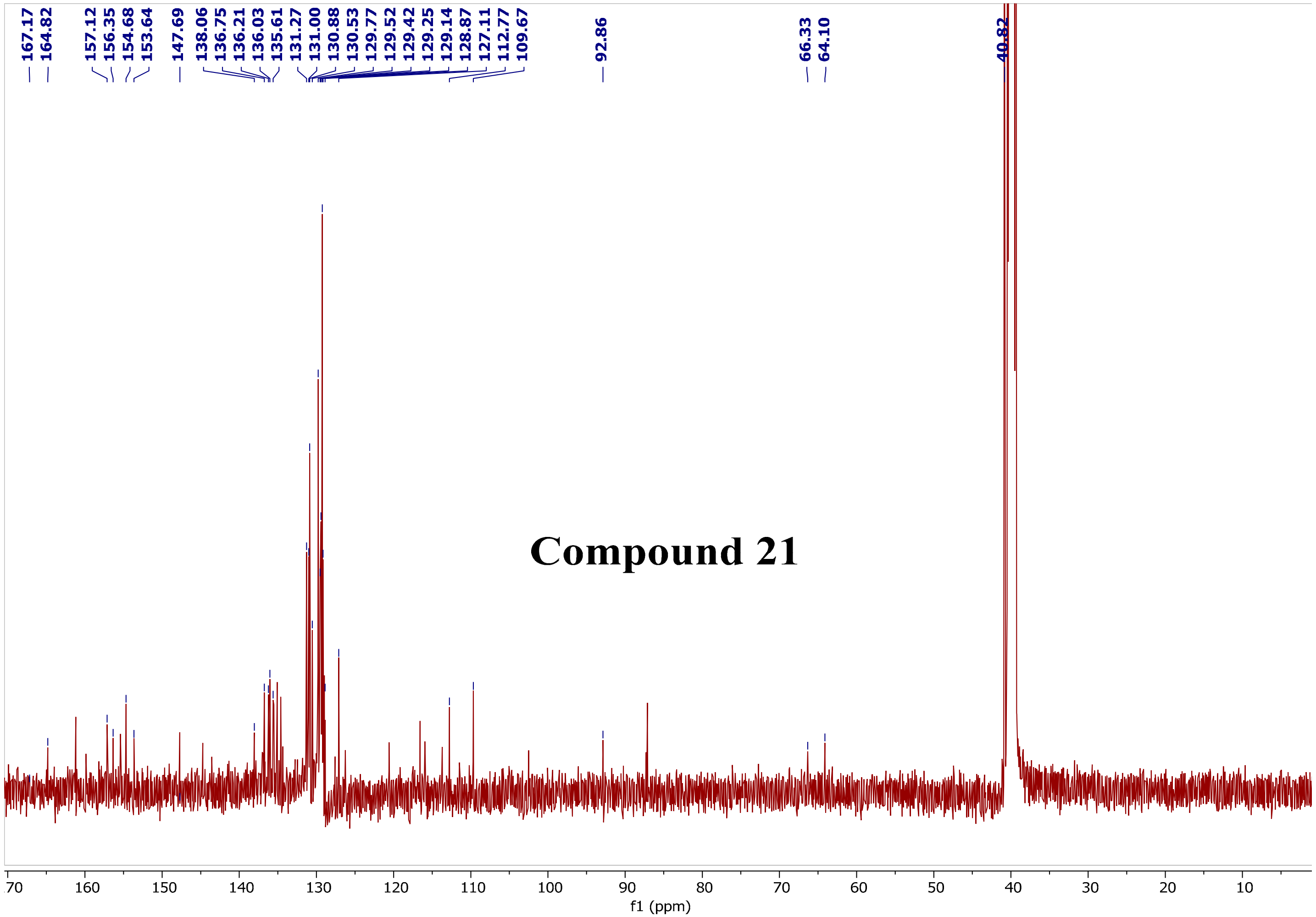


**Figure S2.** $^{13}$C NMR spectrum for compound **21** in DMSO-$d_6$ (125 MHz).

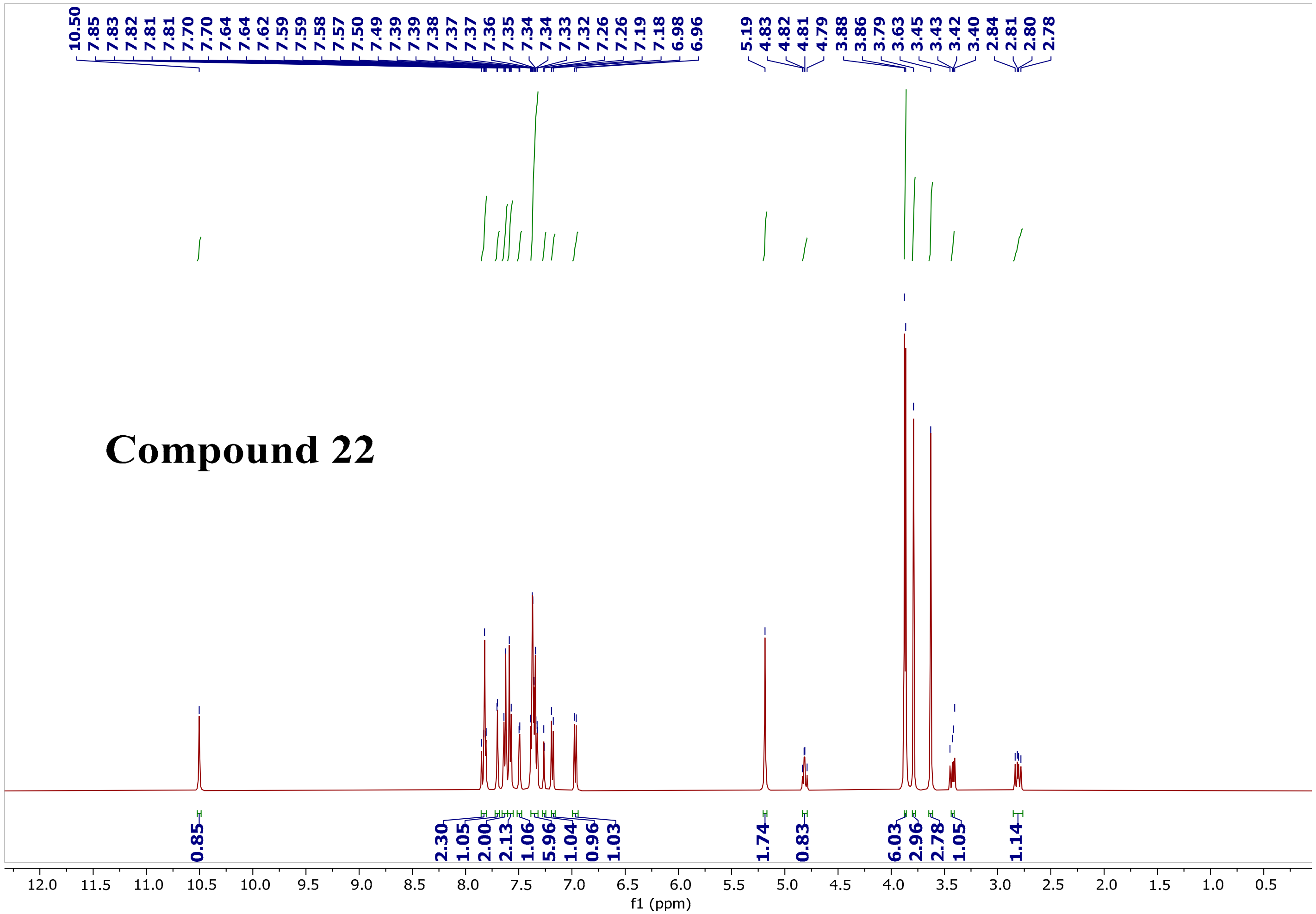


(a)

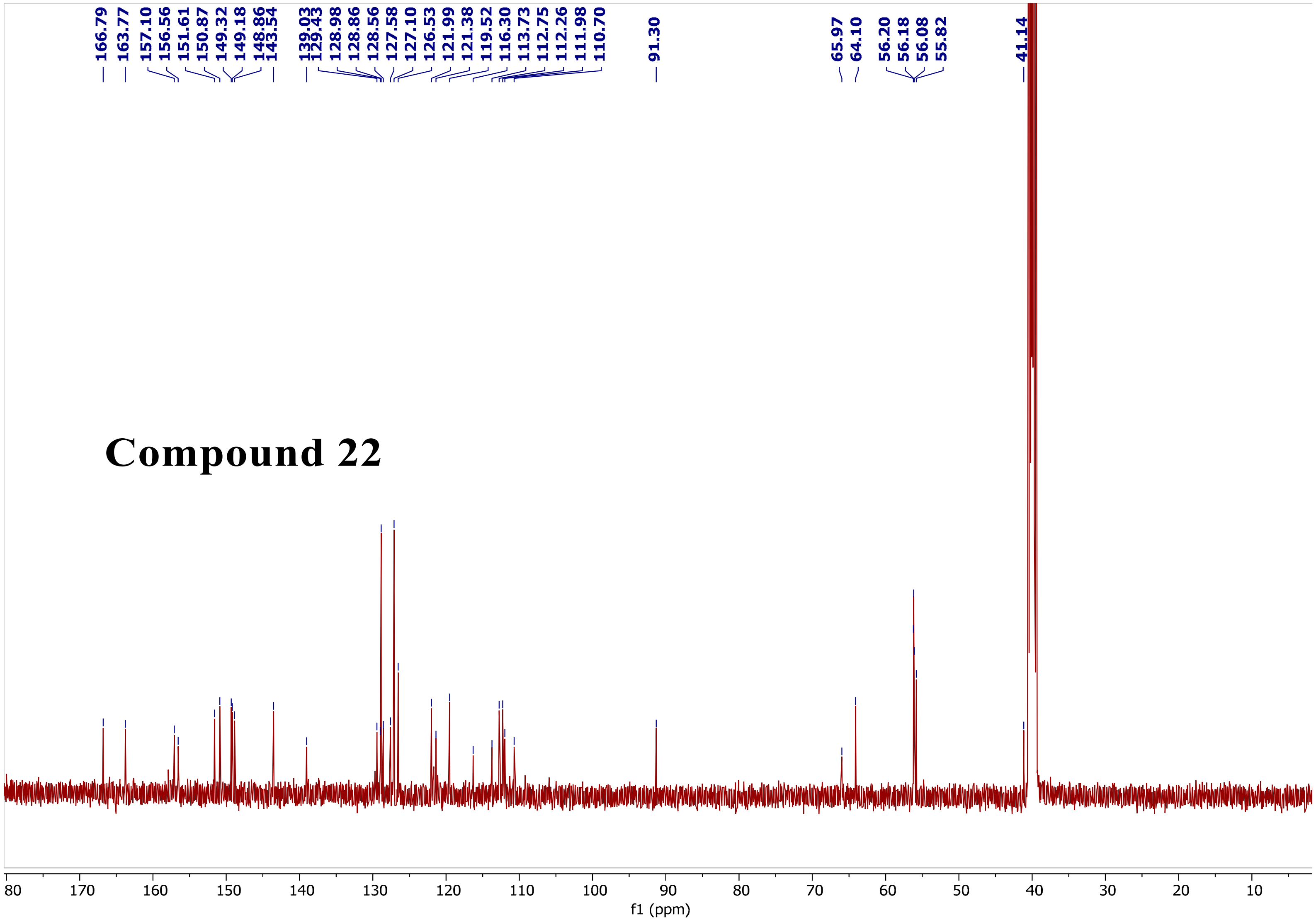


(**b**)

**Figure S3. (a):** $^{1}$H NMR spectrum for compound **22** in DMSO-$d_6$ (500 MHz). (**b**)**:** $^{13}$C NMR spectrum for compound **22** in DMSO-$d_6$ (125 MHz).

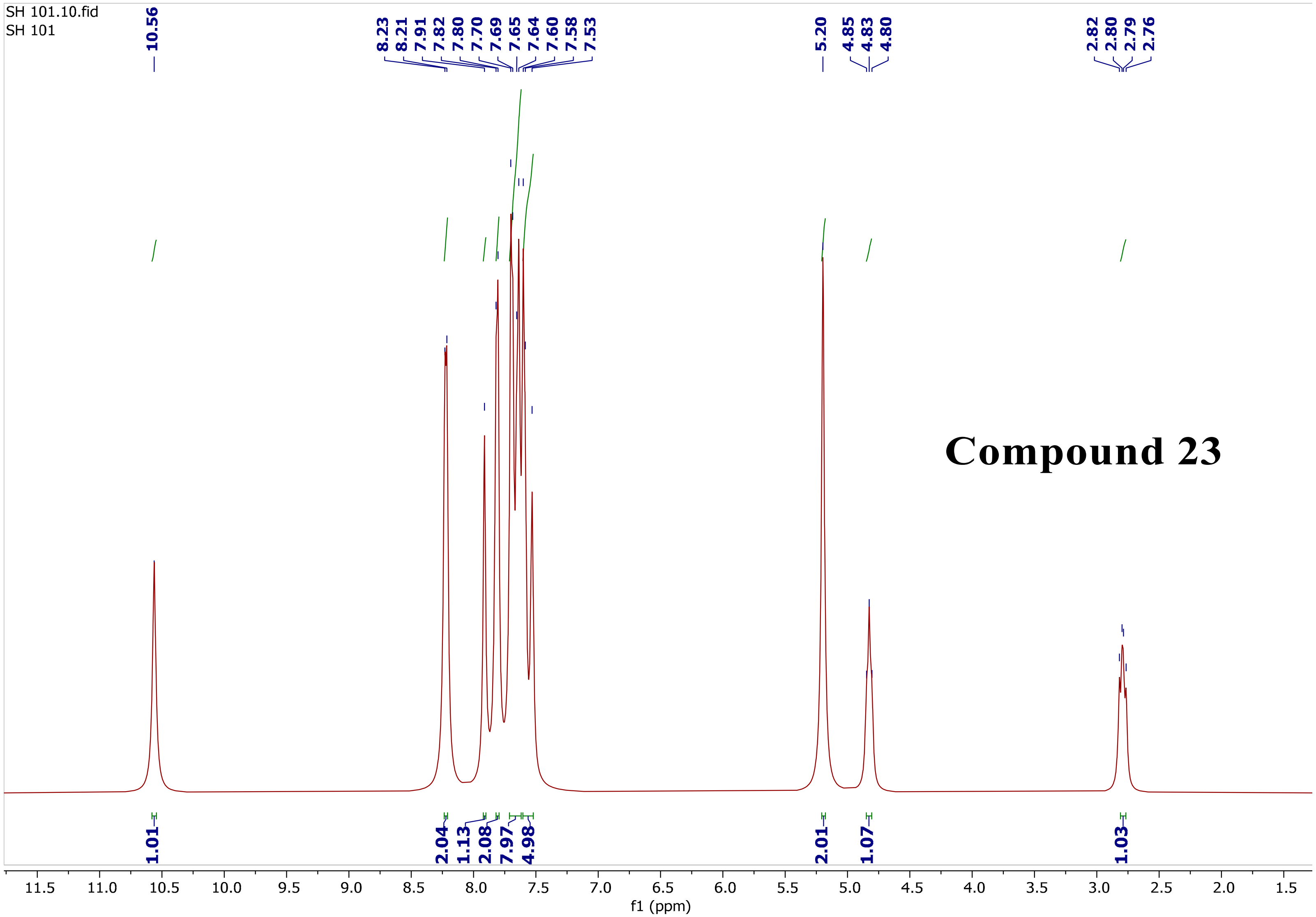


**Figure S4.** $^{1}$H NMR spectrum for compound **23** in DMSO-$d_6$ (500 MHz).

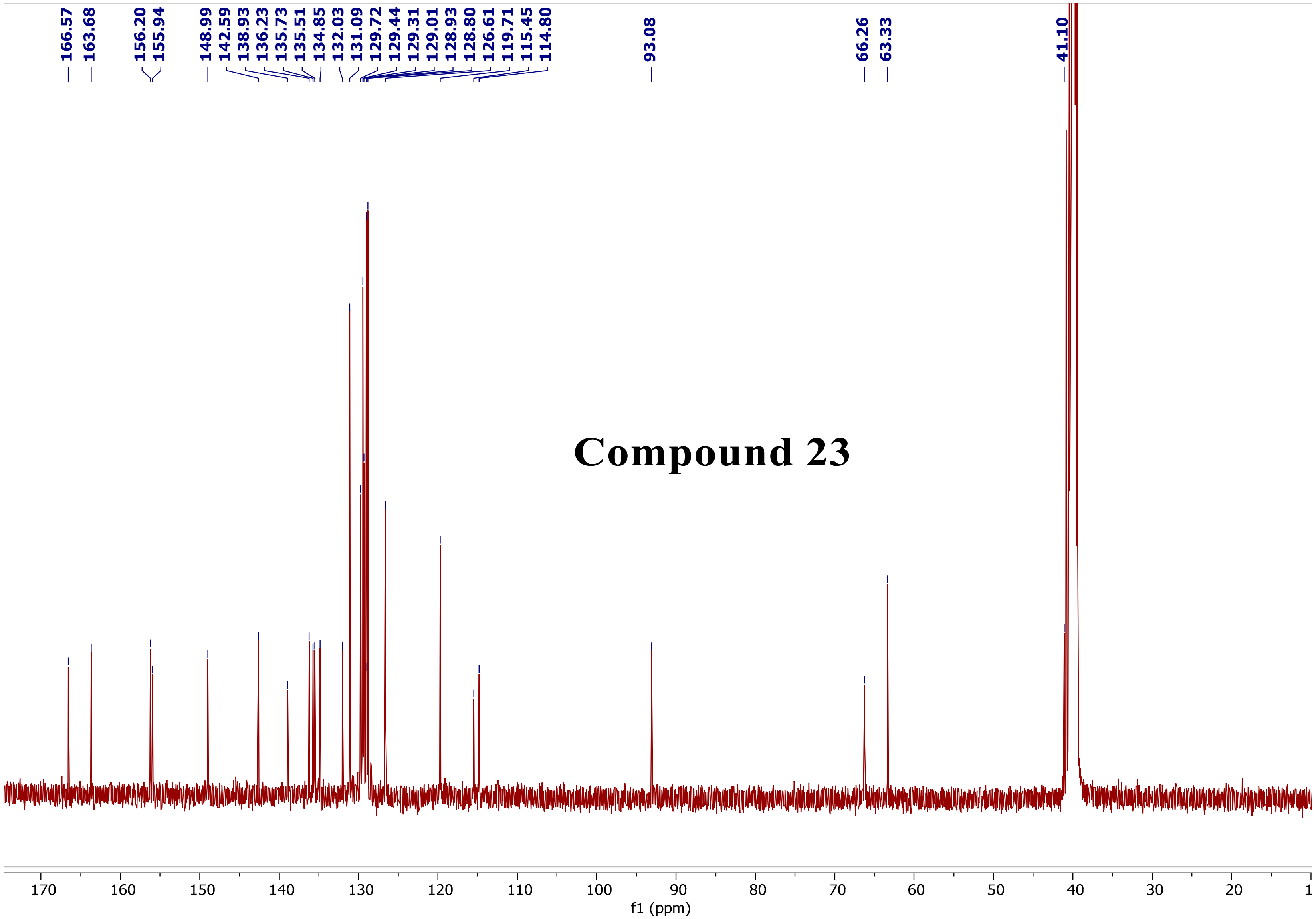


**Figure S5.** $^{13}C$ NMR spectrum for compound **23** in DMSO-$d_6$ (125 MHz).

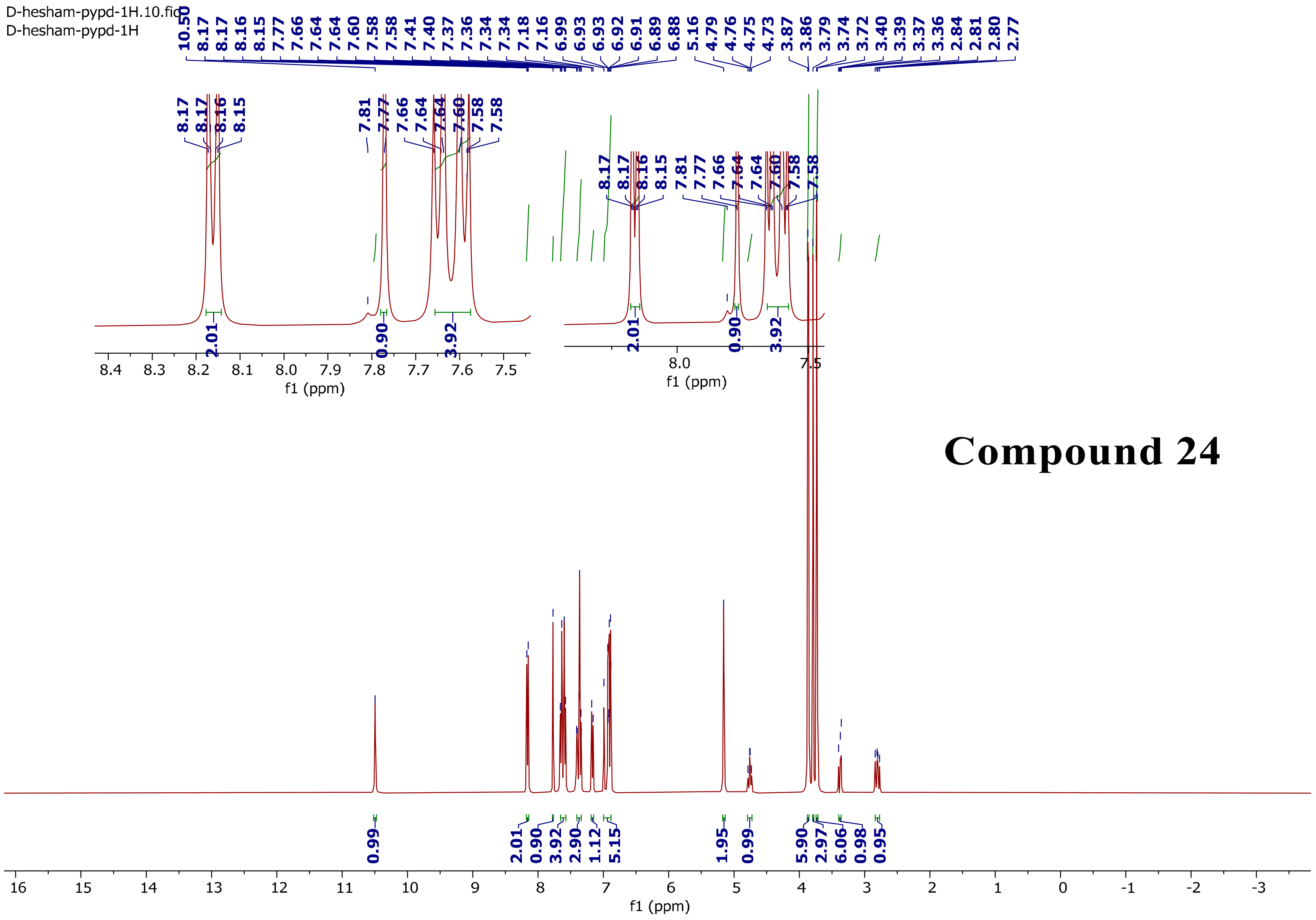


**Figure S6.** ¹H NMR spectrum for compound **24** in DMSO-*d₆* (500 MHz).

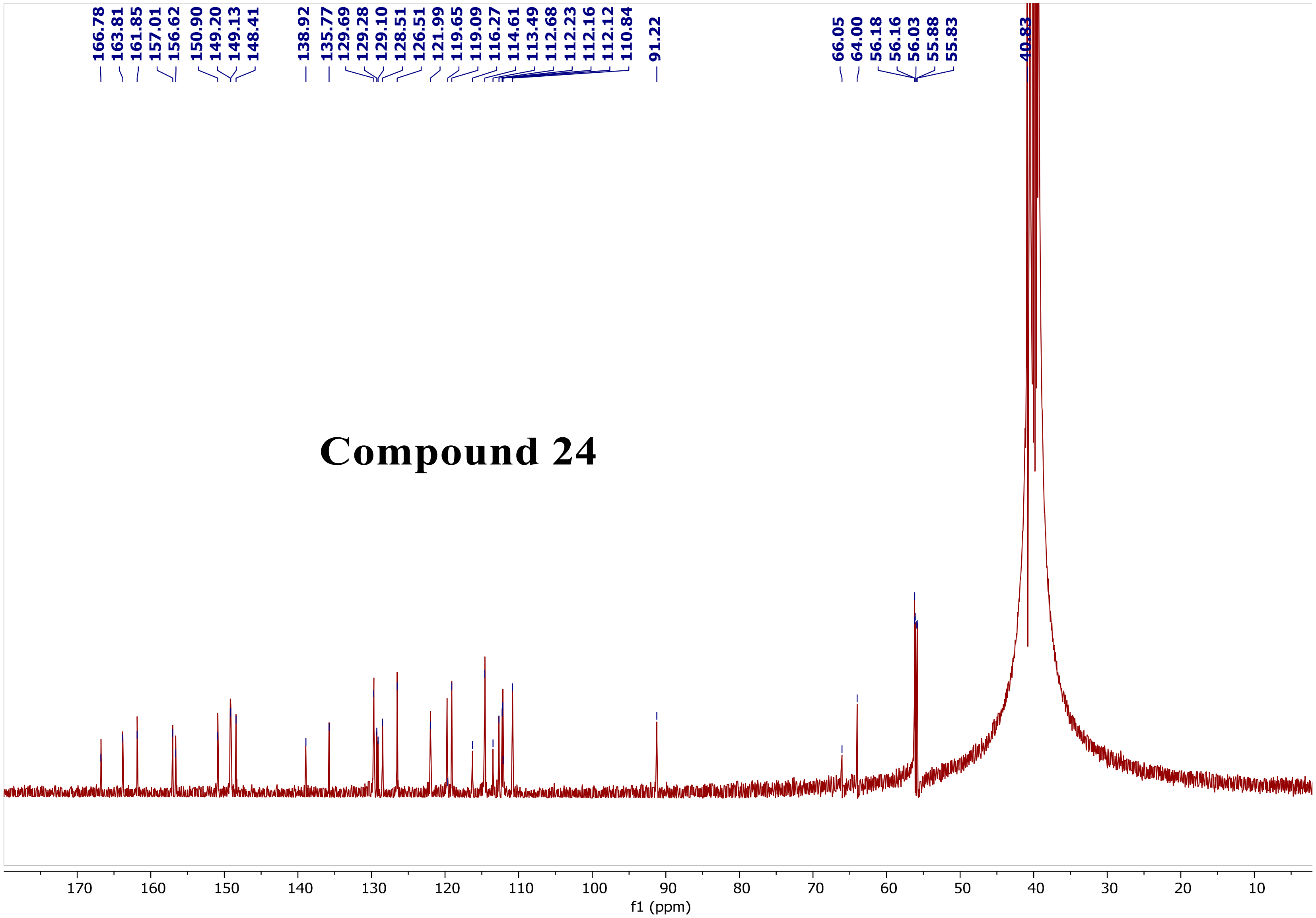


**Figure S7.** $^{13}$C NMR spectrum for compound **24** in DMSO-*d*$_6$ (125 MHz).

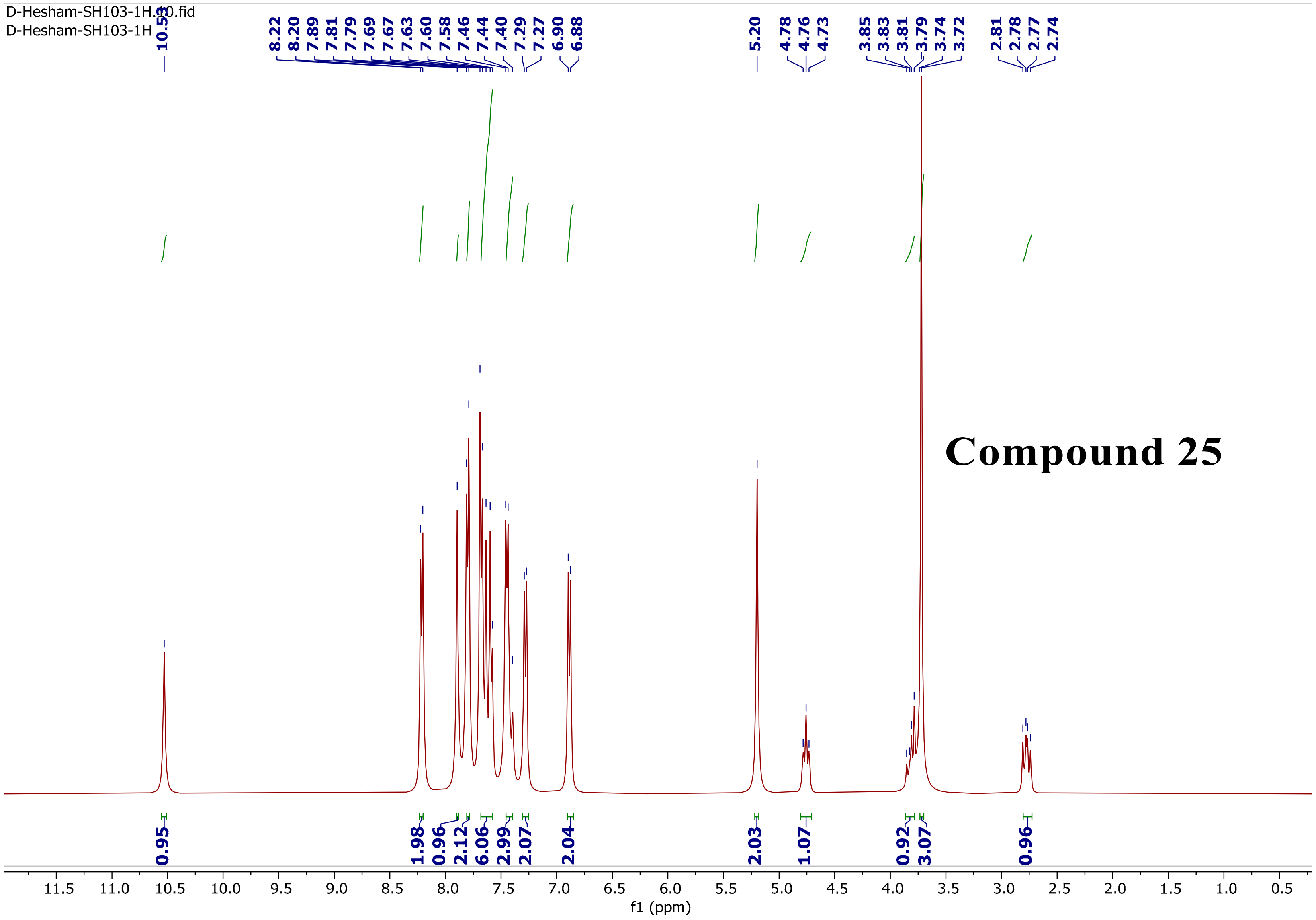


**Figure S8.** $^{1}$H NMR spectrum for compound **24** in DMSO-$d_6$ (500 MHz).

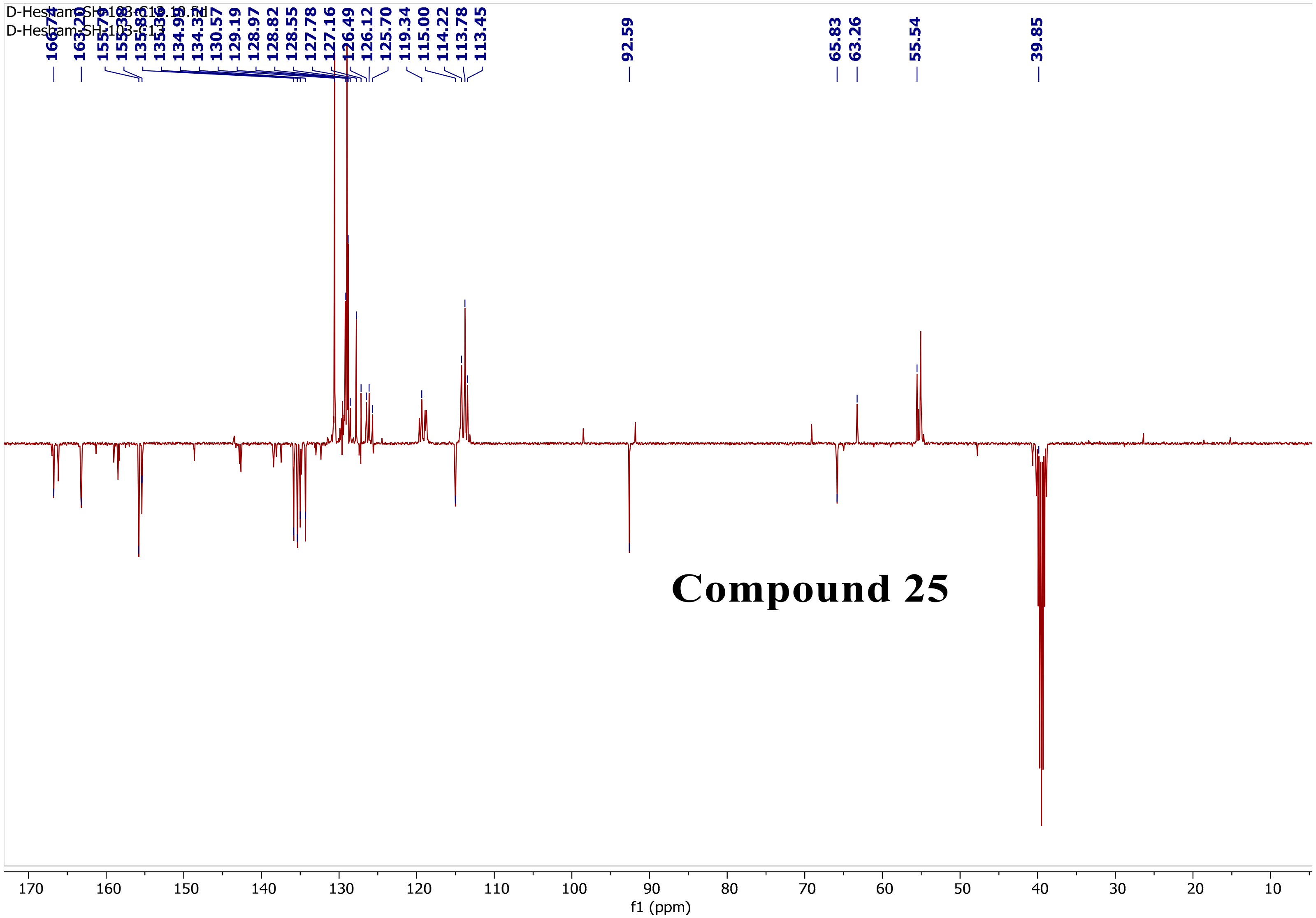


**Figure S9.** DEPT $^{13}$C NMR spectrum for compound **24** in DMSO-$d_6$ (125 MHz).

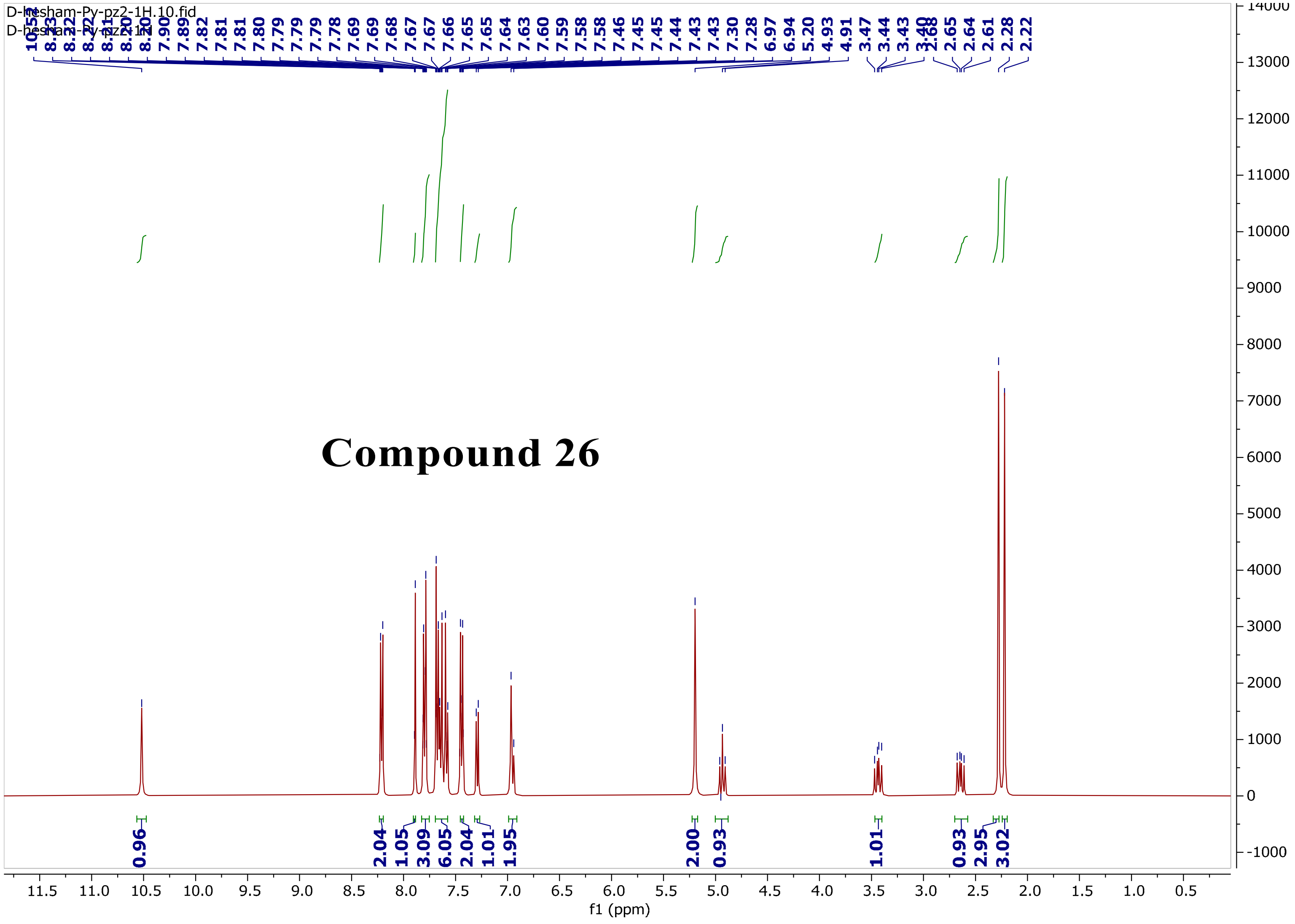


**Figure S10.** $^{1}H$ NMR spectrum for compound **26** in DMSO-$d_6$ (500 MHz).

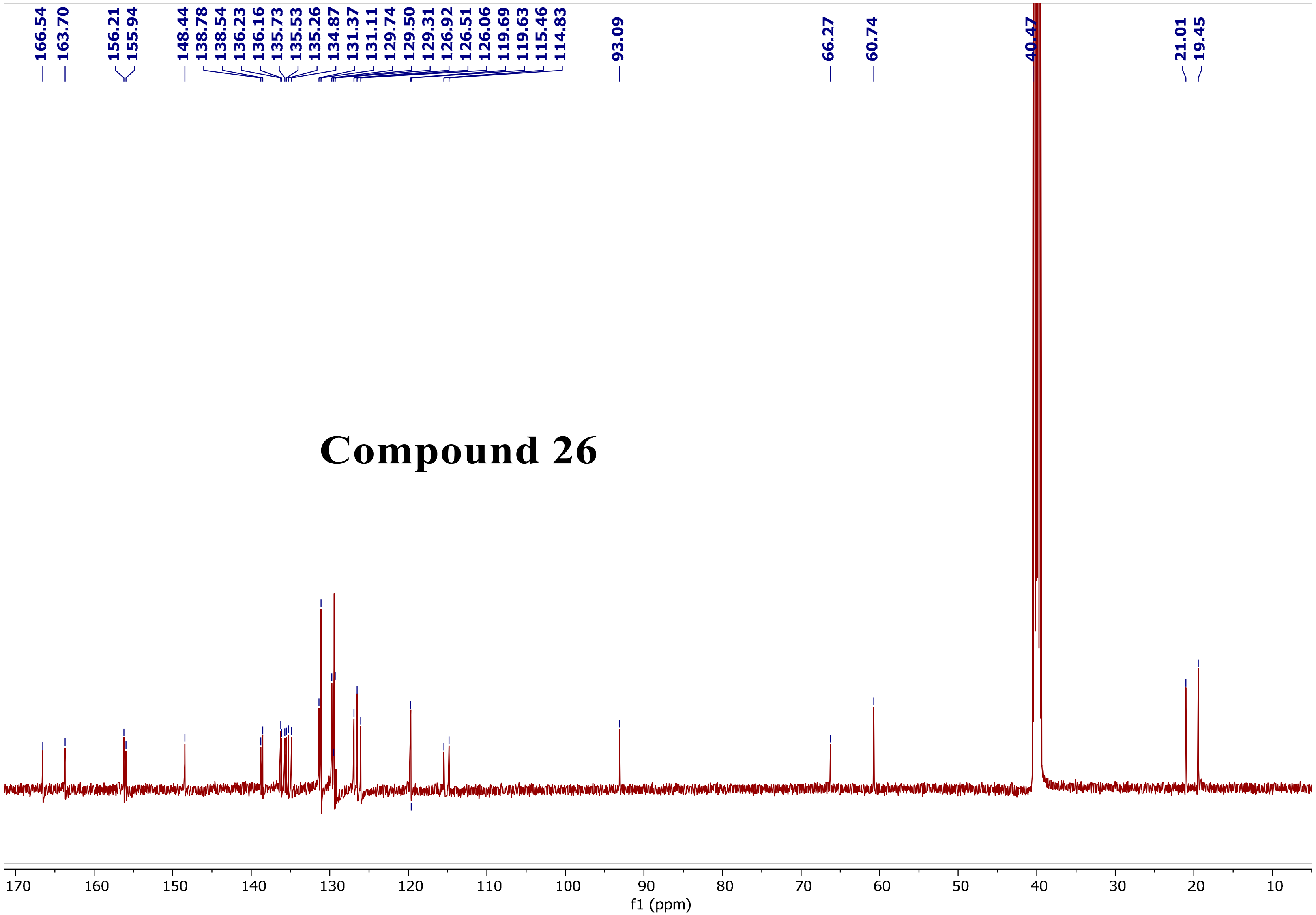


**Figure S11.** $^{13}$C NMR spectrum for compound **26** in DMSO-$d_6$ (125 MHz).

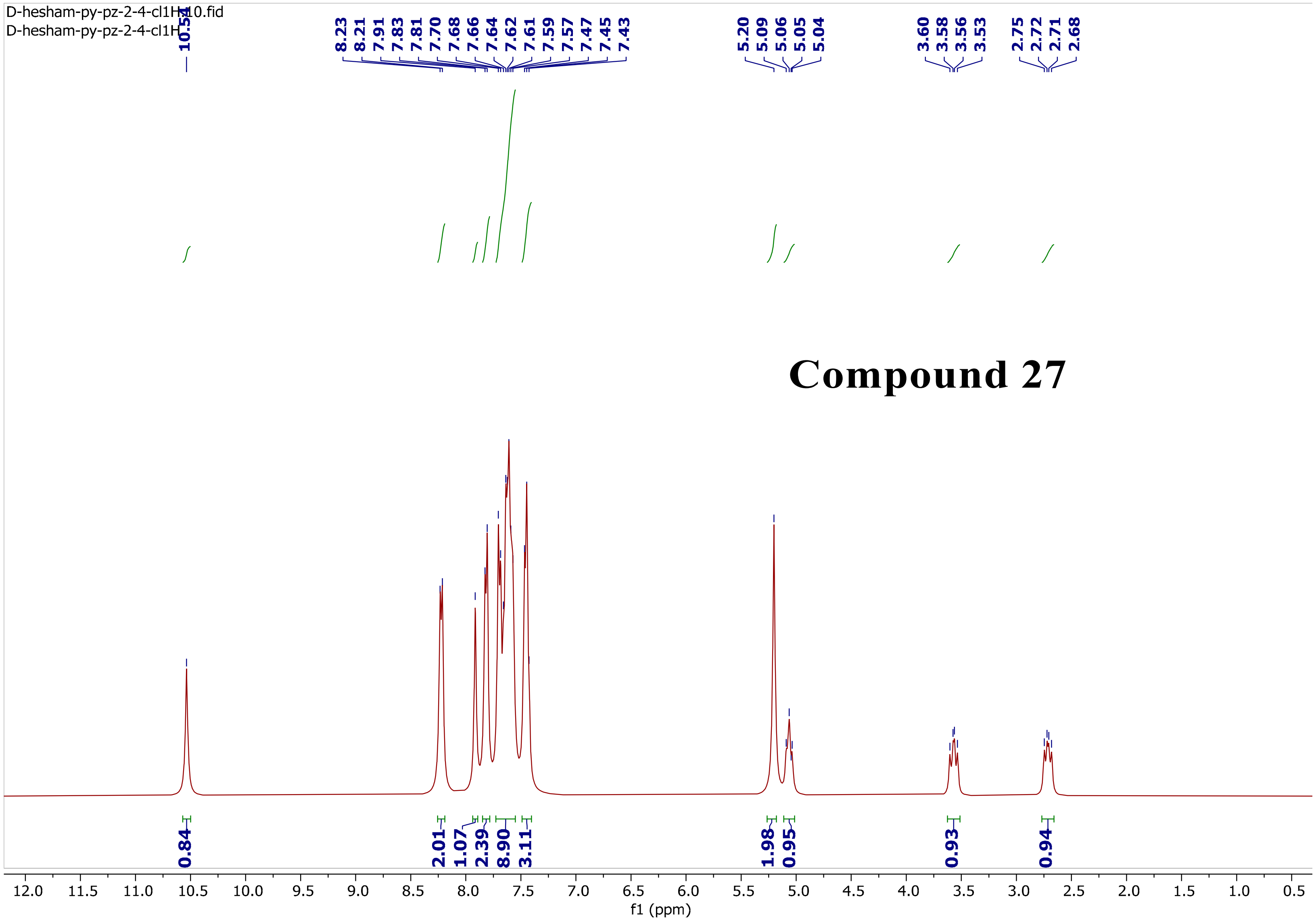


**Figure S12.** $^{1}H$ NMR spectrum for compound **27** in DMSO-$d_6$ (500 MHz).

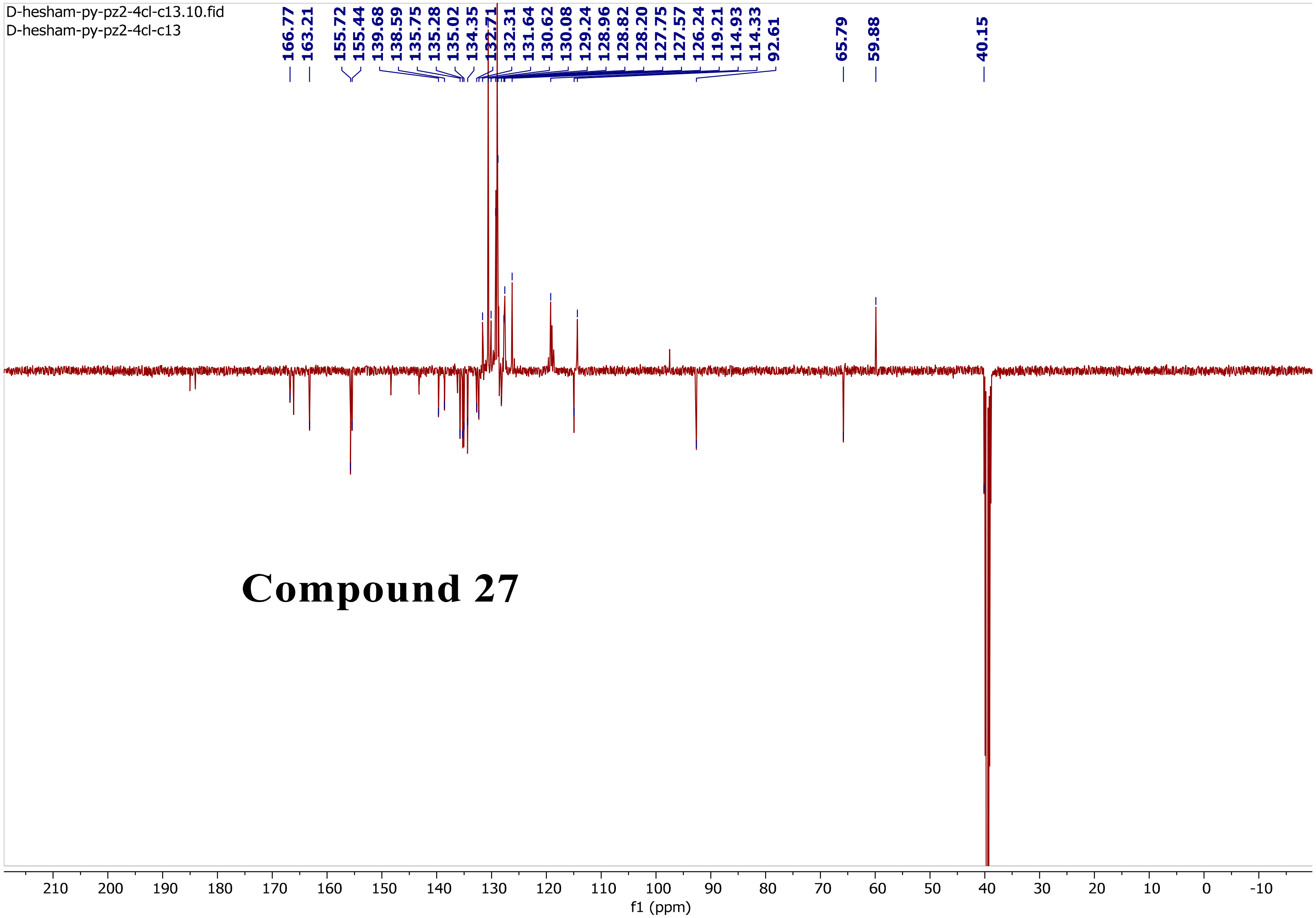


**Figure S13.** DEPT $^{13}$C NMR spectrum for compound **27** in DMSO-$d_6$ (125 MHz).

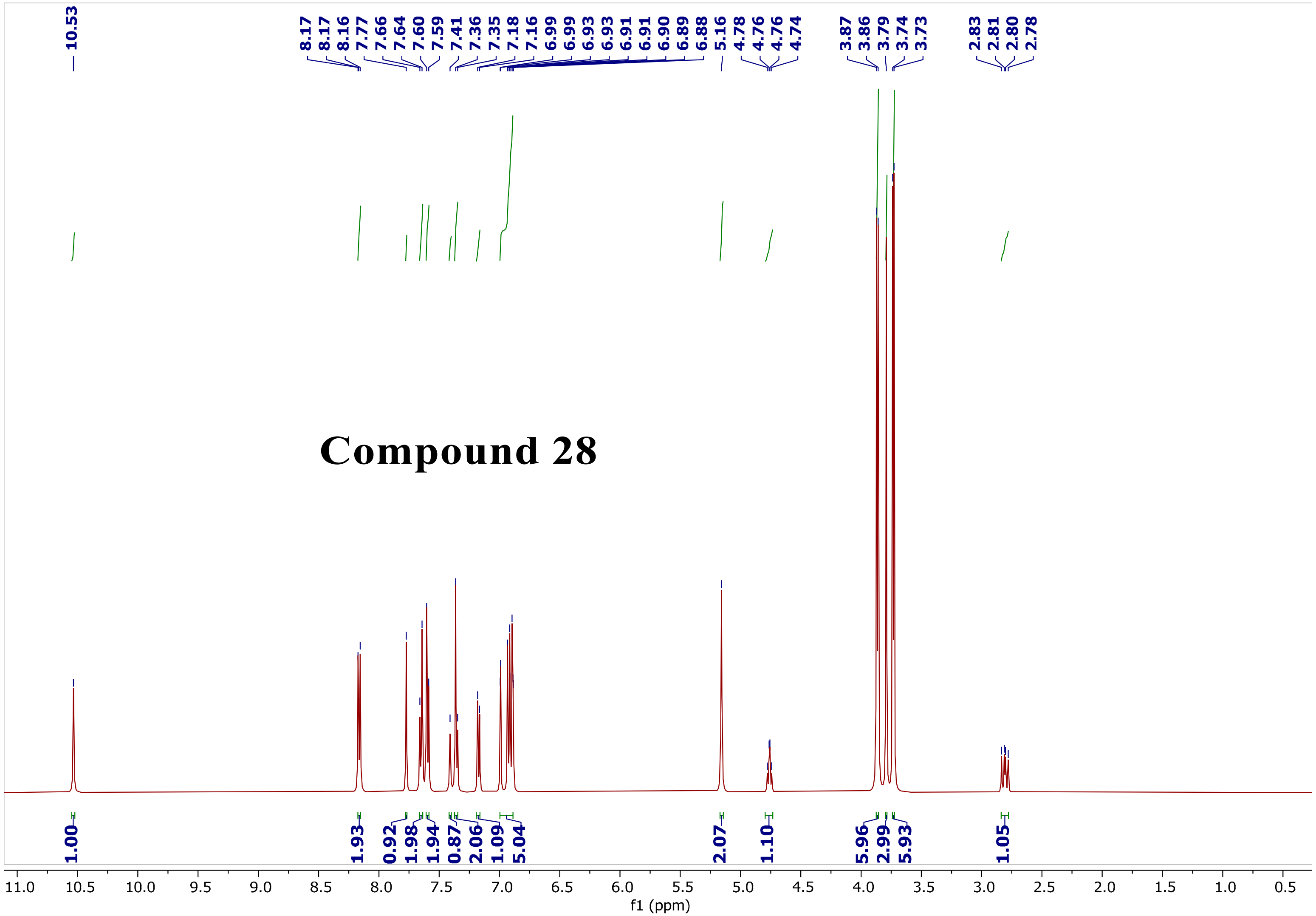


**Figure S14.** $^1$H NMR spectrum for compound **28** in DMSO-$d_6$ (500 MHz).

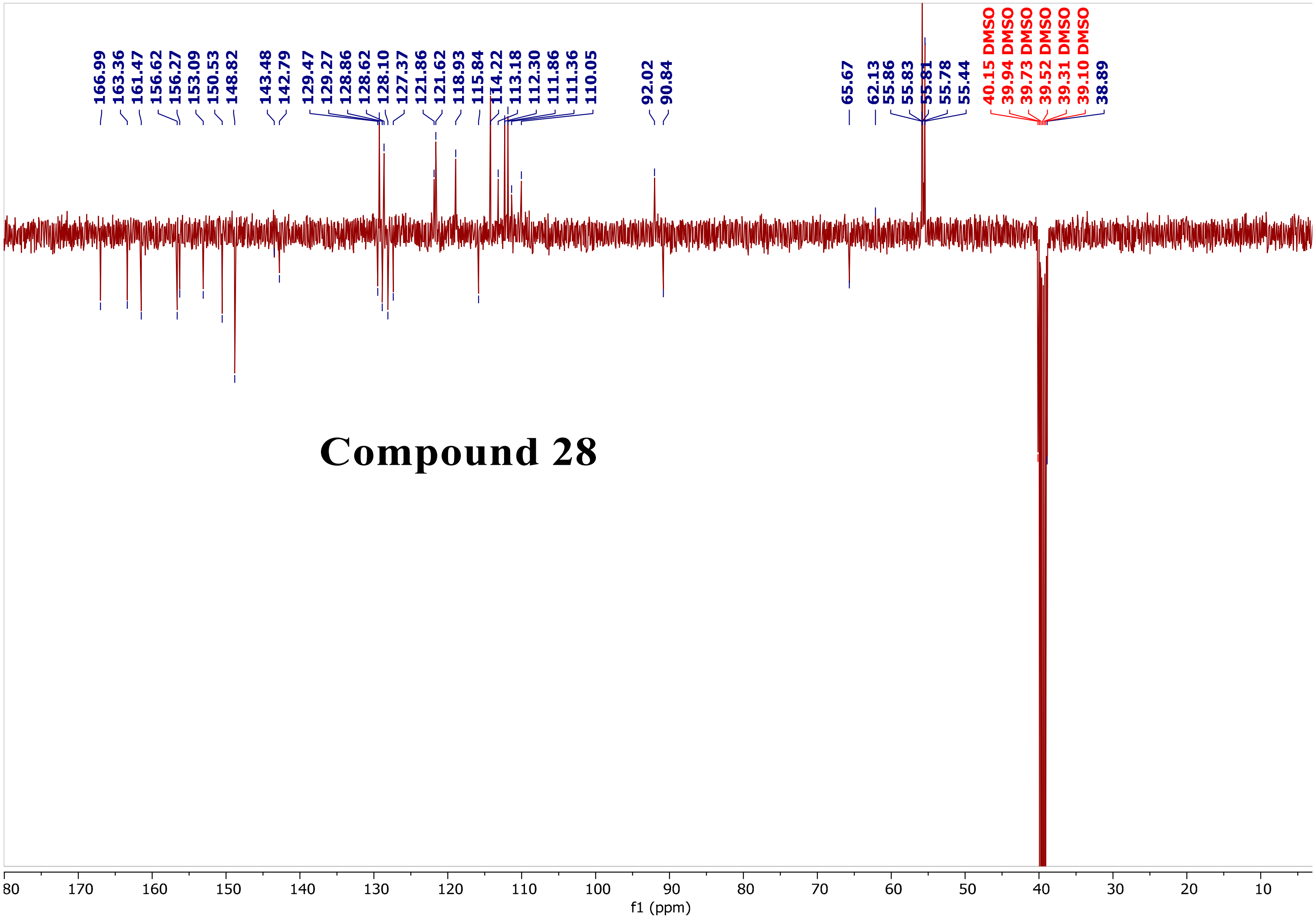


**Figure S15.** DEPT $^{13}$C NMR spectrum for compound **28** in DMSO-$d_6$ (125 MHz).

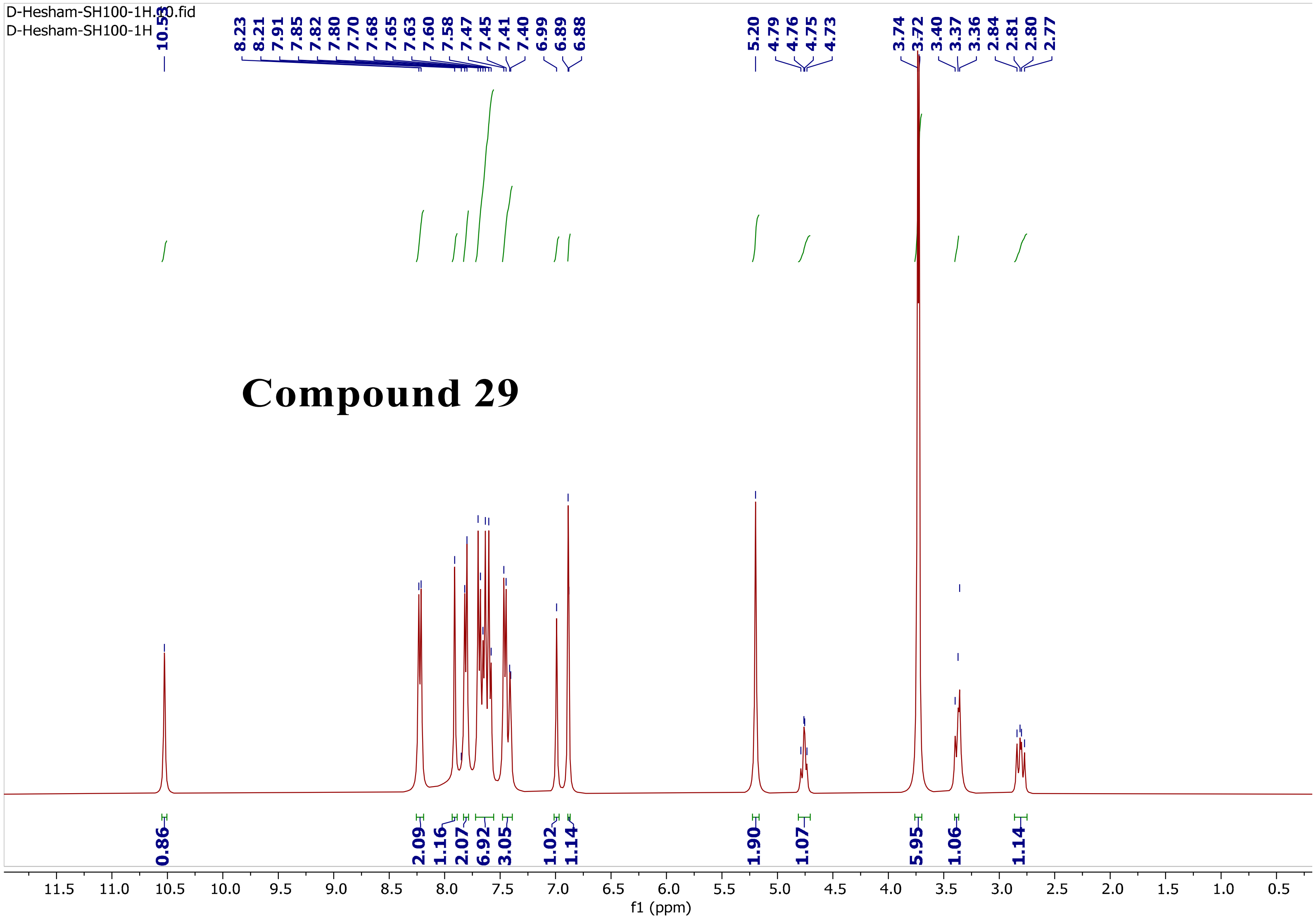


**Figure S16.** $^1$H NMR spectrum for compound **29** in DMSO-$d_6$ (500 MHz).

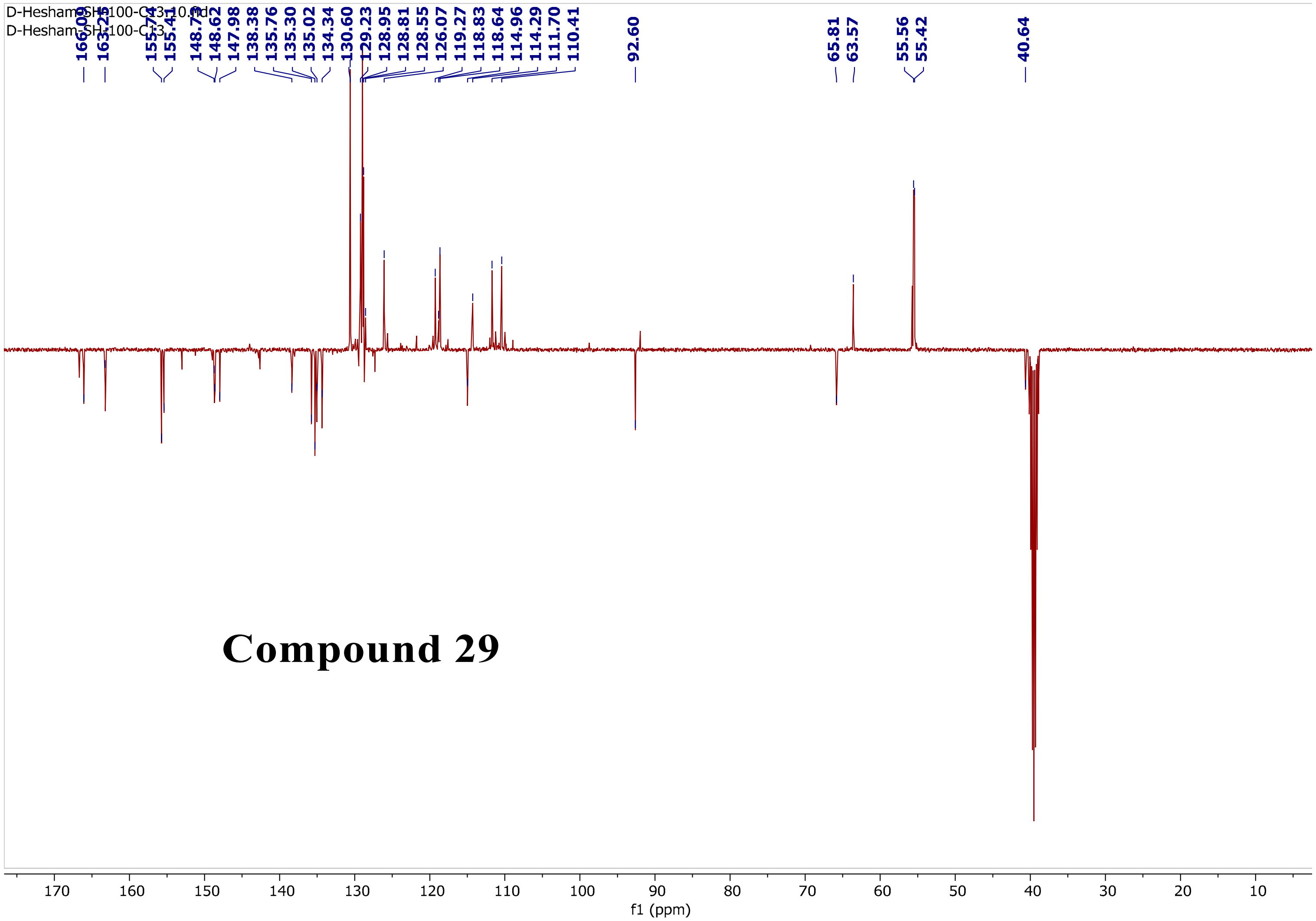


**Figure S17.** DEPT $^{13}$C NMR spectrum for compound **29** in DMSO-$d_6$ (125 MHz).

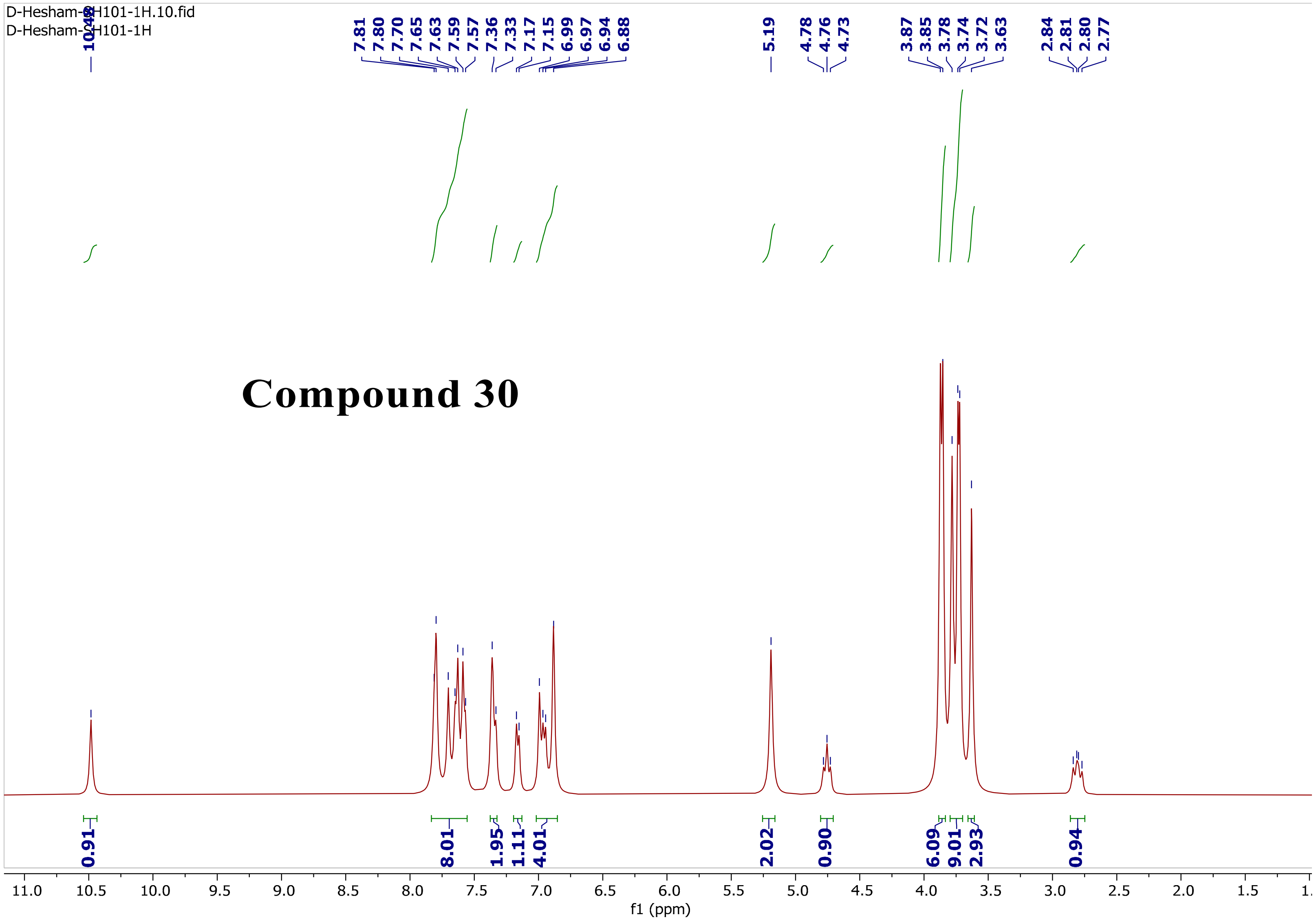


**Figure S18.** $^{1}H$ NMR spectrum for compound **30** in DMSO-$d_6$ (500 MHz).

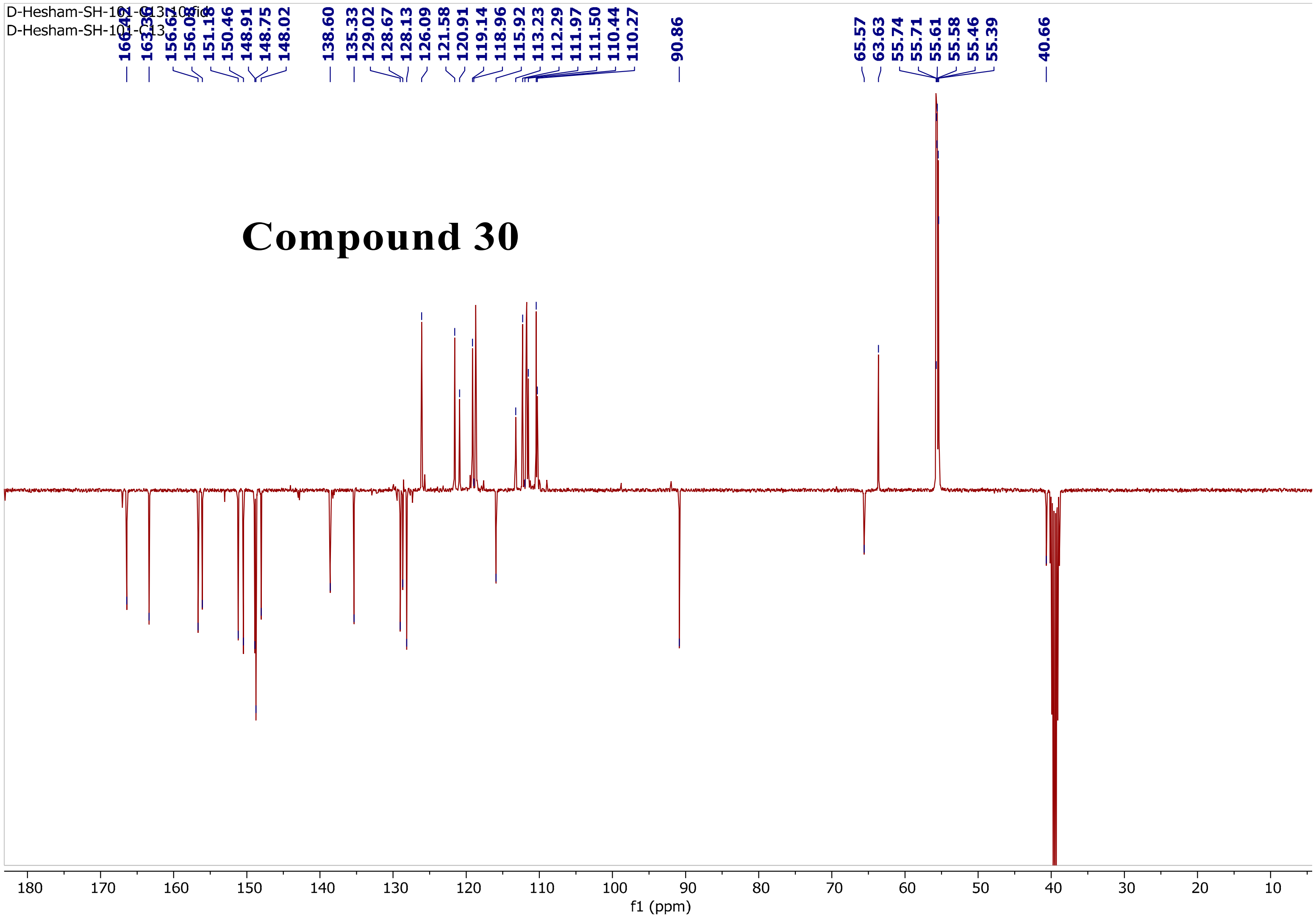


**Figure S19.** DEPT $^{13}C$ NMR spectrum for compound **30** in DMSO-$d_6$ (125 MHz).

# 4. EXPERIMENTAL

## 4.1. Chemistry

### General Details

Chemicals and solvents procured from Alfa Aesar, Sigma Aldrich, and El-Nasr Pharmaceutical Chemical Companies were utilized to synthesize the target compounds. The advancement of the reactions was observed by employing pre-coated TLC plates (Kieselgel 60f254 Merck), and the spots were identified by exposing them to UV light. Uncorrected melting points were measured with Stuart's electrothermal melting point apparatus. A JEOL JNM-GX-600 spectrometer (500 MHz) located at the Faculty of Pharmaceutical Sciences, Umm Al-Qura University in Mecca, Saudi Arabia, and Faculty of Science, Zagazig University in Egypt, was used to perform $^{1}$H-NMR spectra. The chemical shift (δ) in ppm relative to TMS (δ: 0 ppm) was taken as the internal standard, and DMSO-$d_6$ was utilized as the solvent (δ: 2.5 ppm). The signal and coupling constant (J) in Hz were denoted by the following: s for singlet, d for doublet, t for triplet, q for quartet, and m for multiplet. Additionally, $^{13}$C-NMR spectra were recorded on JEOL JNM-GX-150 in Switzerland (125 MHz), with chemical shift (δ) in ppm relative to TMS (δ: 0 ppm) as the internal standard and DMSO-$d_6$ as the solvent (δ: 39.50 ppm). Shimadzu GC/MS-QP5050A at the Regional Centre for Mycology and Biotechnology, Al-Azhar University, was used to conduct elemental analyses, and the results were found to be within ± 0.4% of the theoretical values

### 4.2. Biological Evaluation

#### 4.2.1. Cytotoxic Activity Using MTT Assay and Evaluation of $IC_{50}$

##### 4.2.1.1. MTT Assay

MTT assay was performed to investigate the effect of the synthesized compounds on mammary epithelial cells (MCF-10A). The cells were propagated in medium consisting of Ham's F-12 medium/ Dulbecco's modified Eagle's medium (DMEM) (1:1) supplemented with 10% foetal calf serum, 2 mM glutamine, insulin (10 μg/mL), hydrocortisone (500 ng/mL) and epidermal growth factor (20 ng/mL). Trypsin ethylenediamine tetra acetic acid (EDTA) was used to passage the cells after every 2-3 days. 96-well flat-bottomed cell culture plates were used to seed the cells at a density of 104 cells $mL^{-1}$. The medium was aspirated from all the wells of culture plates after 24 h followed by the addition of synthesized compounds (in 200 μL medium to yield a final concentration of 0.1% (v/v) dimethyl sulfoxide) into individual wells of the plates. Four wells were designated to a single compound. The plates were allowed to incubate at 37°C for 96 h. Afterwards, the medium was aspirated and 3-[4,5-dimethylthiazol-2-yl]-2,5-diphenyltetrazolium bromide (MTT) (0.4 mg/mL) in medium was added to each well and subsequently incubated for 3 h. The medium was aspirated and 150 μL dimethyl sulfoxide (DMSO) was added to each well. The plates were vortexed followed by the measurement of absorbance at 540 nm on a microplate reader. The results were presented as inhibition (%) of proliferation in contrast to controls comprising 0.1% DMSO.

#### 4.2.1.2. Assay for Antiproliferative Effect

To explore the antiproliferative potential of compounds propidium iodide fluorescence assay was performed using different cell lines such as Panc-1 (pancreas cancer cell line), MCF-7 (breast cancer cell line), HT-29 (colon cancer cell line) and A-549 (epithelial cancer cell line), respectively. To calculate the total nuclear DNA, a fluorescent dye (propidium iodide, PI) is used which can attach to the DNA, thus offering a quick and precise technique. PI cannot pass through the cell membrane and its signal intensity can be considered as directly proportional to quantity of cellular DNA. Cells whose cell membranes are damaged or have changed permeability are counted as dead ones. The assay was performed by seeding the cells of different cell lines at a density of 3000-7500 cells/well (in200µl medium) in culture plates followed by incubation for 24h at 37 °C in humidified 5%CO2/95% air atmospheric conditions. The medium was removed; the compounds were added to the plates at 10 µM concentrations (in 0.1% DMSO) in triplicates, followed by incubation for 48h. DMSO (0.1%) was used as control. After incubation, medium was removed followed by the addition of PI (25 µl, 50µg/mL in water/medium) to each well of the plates. At -80 °C, the plates were allowed to freeze for 24 h, followed by thawing at 25oC. A fluorometer (Polar-Star BMG Tech) was used to record the readings at excitation and emission wavelengths of 530 and 620 nm for each well. The percentage cytotoxicity of compounds was calculated using the following formula:

$$\% \text{ Cytotoxicity} = \frac{A_C - A_{TC}}{A_C} \times 100$$

Where A*TC*= Absorbance of treated cells and AC= Absorbance of control. Erlotinib was used as positive control in the assay.

### 4.2.2. EGFR Inhibitory Assay

Baculoviral expression vectors including pBlueBacHis2B and pFASTBacHTc were used separately to clone 1.6 kb cDNA coding for EGFR cytoplasmic domain (EGFR-CD, amino acids 645–1186). 5' upstream to the EGFR sequence comprised a sequence that encoded (His)6. Sf-9 cells were infected for 72h for protein expression. The pellets of Sf-9 cells were solubilized in a buffer containing sodium vanadate (100 μM), aprotinin (10 μg/mL), triton (1%), HEPES buffer(50mM), ammonium molybdate (10 μM), benzamidine HCl (16 μg/mL), NaCl (10 mM),leupeptin (10 μg/mL) and pepstatin (10 μg/mL) at 0°C for 20 min at pH 7.4, followed by centrifugation for 20 min. To eliminate the nonspecifically bound material, a Ni-NTA super flow packed column was used to pass through and wash the crude extract supernatant first with 10mM and then with 100 mM imidazole. Histidine-linked proteins were first eluted with 250 and then with 500 mM imidazole subsequent to dialysis against NaCl (50 mM), HEPES (20 mM), glycerol (10%) and 1 μg/mL each of aprotinin, leupeptin and pepstatin for 120 min. The purification was performed either at 4 °C or on ice. To record autophosphorylation level, EGFR kinase assay was carried out on the basis of DELFIA/Time-Resolved Fluorometry. The compounds were first dissolved in DMSO absolute,

subsequent to dilution to appropriate concentration using HEPES (25 mM) at pH 7.4. Each compound (10 µL) was incubated with recombinant enzyme (10 µL, 5 ng for EGFR, 1:80 dilution in 100 mM HEPES) for 10 min at 25$^{o}$C, subsequent to the addition of 5X buffer (10 µL, containing 2 mM MnCl2, 100 µM Na3VO4, 20 mM HEPES and 1 mM DTT) and ATP-MgCl2 (20 µL, containing 0.1 mM ATP and 50 mM MgCl2) and incubation for 1h. The negative and positive controls were included in each plate by the incubation of enzyme either with or without ATP-MgCl2. The liquid was removed after incubation and the plates were washed thrice using wash buffer. Europium-tagged antiphosphotyrosine antibody (75 µL, 400 ng) was added to each well followed by incubation of 1h and then washing of the plates using buffer. The enhancement solution was added to each well and the signal was recorded at excitation and emission wavelengths of 340 at 615 nm. The autophosphorylation percentage inhibition by compounds was calculated using the following equation:

$$100\% - [(negative\ control)/(positive\ control) - (negative\ control)]$$

Using the curves of percentage inhibition of eight concentrations of each compound, IC50 was calculated. Majority of signals detected by antiphosphotyrosine antibody were from EGFR because the enzyme preparation contained low impurities.

### 4.2.3. BRAF$^{V600E}$ Inhibitory Assay

$V^{600E}$ mutant BRAF kinase assay was performed to investigate the activity of tested compounds against BRAF. Mouse full-length GST-tagged BRAF$^{V600E}$ (7.5 ng, Invitrogen, PV3849) was pre-incubated with drug (1 μL) and assay dilution buffer (4 μL) for 60 min at 25°C. In assay dilution buffer, a solution (5 μL) containing $MgCl_2$ (30 mM), ATP (200 μM), recombinant human full length (200 ng) and *N*-terminal His-tagged MEK1 (Invitrogen) was added to start the assay, subsequent to incubation for 25 min at 25°C. The assay was stopped using 5X protein denaturing buffer (LDS) solution (5 μL). To further denature the protein, heat (70° C) was applied for 5 min. 4-12% precast NuPage gel plates (Invitrogen) were used to carry out electrophoresis (at 200 V). 10 μL of each reaction was loaded into the precast plates and electrophoresis was allowed to proceed. After completion of electrophoresis, the front part of the precast gel plate (holding hot ATP) was cut and afterwards cast-off. The dried gel was developed using a phosphor screen. A reaction without active enzyme was used as negative control while that containing no inhibitor served as positive control. To study the effect of compounds on cell-based pERK1/2 activity in cancer cells, commercially available ELISA kits (Invitrogen) were used according to manufacturer's instructions.

#### 4.2.4. Caspase-3 Activation Assay

Allow all reagents to reach room temperature before use. Gently mix all liquid reagents prior to use. Determine the number of 8-well strips needed for the assay. Insert these in the frame(s) for current use. Add 100 µl of the *Standard Diluent Buffer* to the zero standard wells. Well(s) reserved for chromogen blank should be left empty. Add 100 µl of standards and controls or diluted samples to the appropriate microtiter wells. The sample dilution chosen should be optimized for each experimental system. Tap gently on side of plate to mix. Cover wells with *plate cover* and incubate for 2 hours at room temperature. Thoroughly aspirate or decant solution from wells and discard the liquid, Wash wells 4 times. Pipette 100 µl of *Caspase-3 (Active) Detection Antibod*y solution into each well except the chromogen blank(s). Tap gently on the side of the plate to mix. Cover plate with *plate cover* and incubate for 1 hour at room temperature. Thoroughly aspirate or decant solution from wells and discard the liquid, Wash wells 4 times. Add 100 µl Anti-Rabbit IgG HRP Working Solution to each well except the chromogen blank(s). Prepare the working dilution as described in Preparing IgG HRP. Cover wells with the *plate cover* and incubate for 30 minutes at room temperature. Thoroughly aspirate or decant solution from wells and discard the liquid. Wash wells 4 times. Add 100 µl of *Stabilized Chromogen* to each well. The liquid in the wells will begin to turn blue. Incubate for 30 minutes at room temperature and in the dark. The incubation time for chromogen substrate is often determined by the microtiter plate reader used. Many plate readers have the capacity to record a maximum optical density (O.D.) of 2.0. The O.D. values should be monitored, and the substrate reaction stopped before the O.D. of the positive wells

exceeds the limits of the instrument. The O.D. values at 450 nm can only be read after the *Stop Solution* has been added to each well. If using a reader that records only to 2.0 O.D., stopping the assay after 20 to 25 minutes is suggested. Add 100 µl of *Stop Solution* to each well. Tap side of plate gently to mix. The solution in the wells should change from blue to yellow. Read the absorbance of each well at 450 nm having blanked the plate reader against a chromogen blank composed of 100 µl each of *Stabilized Chromogen* and *Stop Solution*. Read the plate within 2 hours after adding the *Stop Solution*. Use a curve fitting software to generate the standard curve. A four-parameter algorithm provides the best standard curve fit. Read the concentrations for unknown samples and controls from the standard curve. Multiply value(s) obtained for sample(s) by the appropriate dilution factor to correct for the dilution in step 3. Samples producing signals greater than that of the highest standard should be diluted in *Standard Diluent Buffer* and reanalyzed.

**4.2.5. Caspase-8 Activation Assay**

Cells were obtained from American Type Culture Collection, cells were grown in RPMI 1640 containing 10% fetal bovine serum at 37°C, stimulated with the compounds to be tested for caspase8, and lysed with Cell Extraction Buffer. This lysate was diluted in Standard Diluent Buffer over the range of the assay and measured for human active caspase-8 content. (*Cells are Plated in a density of 1.2 – 1.8 × 10,000 cells/well in a volume of 100µl complete growth medium + 100 ul of the tested compound per well in a 96-well plate for 24 hours before the enzyme assay for Tubulin*.). The absorbance of each microwell was read on a spectro-photometer at 450 nm. A standard curve is prepared from 7human Caspase-8 standard dilutions and human Caspase-8 concentration determined.

#### 4.2.6. Bax Activation Assay

Bring all reagents, except the human Bax-$\alpha$ Standard, to room temperature for at least 30 minutes prior to opening. The human Bax-$\alpha$ Standard solution should not be left at room temperature for more than 10 minutes. All standards, controls and samples should be run in duplicate. Refer to the Assay Layout Sheet to determine the number of wells to be used and put any remaining wells with the desiccant back into the pouch and seal the ziploc. Store unused wells at 4 °C. Pipet 100 μL of Assay Buffer into the S0 (0 pg/mL standard) wells. Pipet 100 μL of Standards #1 through #6 into the appropriate wells. Pipet 100 μL of the Samples into the appropriate wells. Tap the plate gently to mix the contents. Seal the plate and incubate at room temperature on a plate shaker for 1 hour at ~500 rpm. Empty the contents of the wells and wash by adding 400 μL of wash solution to every well. Repeat the wash 4 more times for a total of 5 washes. After the final wash, empty or aspirate the wells and firmly tap the plate on a lint free paper towel to remove any remaining wash buffer. Pipet 100 μL of yellow Antibody into each well, except the Blank. Seal the plate and incubate at room temperature on a plate shaker for 1 hour at ~500 rpm. Empty the contents of the wells and wash by adding 400 μL of wash solution to every well. Repeat the wash 4 more times for a total of **5** washes. After the final wash, empty or aspirate the wells and firmly tap the plate on a lint free paper towel to remove any remaining wash buffer. Add 100 μL of blue Conjugate to each well, except the Blank. Seal the plate and incubate at room temperature on a plate shaker for 30 minutes at ~500 rpm. Empty the contents of the wells and wash by adding 400 μL of wash solution to every well. Repeat the wash 4 more times for a total of **5 washes**. After the final wash, empty or aspirate the wells and firmly tap the plate on a lint free paper towel to remove any remaining wash buffer.

Pipet 100 μL of Substrate Solution into each well. Incubate for 30 minutes at room temperature on a plate shaker at ~500 rpm. Pipet 100 μL Stop Solution to each well. Blank the plate reader against the Blank wells, read the optical density at 450 nm. Calculate the average net Optical Density (OD) bound for each standard and sample by subtracting the average Blank OD from the average OD for each standard and sample. Using linear graph paper, plot the Average Net OD for each standard versus Bax concentration in each standard. Approximate a straight line through the points. The concentration of Bax in the unknowns can be determined by interpolation.

**4.2.7. Bcl-2inhibition Assay**

Mix all reagents thoroughly without foaming before use. Wash the microwells twice with approximately 300 μL Wash Buffer per well with thorough aspiration of microwell contents between washes. Take caution not to scratch the surface of the microwells. After the last wash, empty the wells and tap microwell strips on absorbent pad or paper towel to remove excess Wash Buffer. Use the microwell strips immediately after washing or place upside down on a wet absorbent paper for not longer than 15 minutes. Do not allow wells to dry. Add 100 μL of Sample Diluent in duplicate to all standard wells and to the blank wells. Prepare standard (1:2 dilution) in duplicate ranging from 32 ng/mL to 0.5 ng/mL. Add 100 μL of Sample Diluent, in duplicate, to the blank wells. Add 80 μL of Sample Diluent, in duplicate, to the sample wells. Add 20 μL of each Sample, in duplicate, to the designated wells. Add 50 μL of diluted biotin-conjugate to all wells, including the blank wells. Cover with a plate cover and incubate at room temperature, on a microplate shaker at 100 rpm if available, for 2 hours. Remove plate cover and empty the wells. Wash microwell strips 3 times as described in step 2. Add 100 μL of diluted

Streptavidin-HRP to all wells, including the blank wells. Cover with a plate cover and incubate at room temperature, on a microplate shaker at 100 rpm if available, for 1 hour. Remove plate cover and empty the wells. Wash microwell strips 3 times as described in step 2. Proceed to the next step. Pipette 100 µl of mixed TMB Substrate Solution to all wells, including the blanks. Incubate the microwell strips at room temperature (18° to 25°C) for about 15 minutes, if available on a rotator set at 100 rpm. Avoid direct exposure to intense light. The point at which the substrate reaction is stopped, is often determined by the ELISA reader. Many ELISA readers record absorbance only up to 2.0 O.D. Therefore, the color development within individual microwells must be watched by the person running the assay and the substrate reaction stopped before positive wells are no longer properly detectable. Stop the enzyme reaction by quickly pipetting 100 µL of Stop Solution into each well, including the blank wells. It is important that the Stop Solution is spread quickly and uniformly throughout the microwells to completely inactivate the enzyme. Results must be read immediately after the Stop Solution is added or within one hour if the microwell strips are stored at 2 - 8°C in the dark. Read absorbance of each microwell on a spectrophotometer using 450 nm as the primary wavelength.

### 4.3. Statistical Analysis

Computerized Prism 5 program was used to statistically analyzed data using one-way ANOVA test followed by Tukey's as post ANOVA for multiple comparison at $P \leq .05$. Data were presented as mean ± SEM.
